# Intraspecific variation in masting across climate gradients is inconsistent with the environmental stress hypothesis

**DOI:** 10.1002/ecy.70076

**Published:** 2025-04-03

**Authors:** Jessie J. Foest, Thomas Caignard, Ian S. Pearse, Michał Bogdziewicz, Andrew Hacket‐Pain

**Affiliations:** ^1^ Geography and Planning, School of Environmental Sciences University of Liverpool Merseyside Liverpool UK; ^2^ Forest Biology Center Faculty of Biology, Adam Mickiewicz University Poznań Wielkopolska Poland; ^3^ INRAE, BIOGECO Université de Bordeaux Pessac Gironde France; ^4^ U.S. Geological Survey Fort Collins Science Center Fort Collins Colorado USA

**Keywords:** alternate bearing, climate gradients, climate marginality, intraspecific variation, mast seeding, masting, reproductive strategy

## Abstract

Year‐to‐year variation in seed crop size (i.e., masting) varies strongly among populations of the same species. Understanding what causes this variation is vital, as masting affects the ability of tree species to regenerate and determines the population dynamics of a wide variety of animals. It is commonly thought that environmental stress is a key driver of masting variability. The environmental stress hypothesis posits that more marginal conditions increase the strength of masting. Using 437 time series from 19 tree species, we find that this hypothesis fails to fully explain how masting varies across marginality gradients. We expected higher interannual variation and less frequent masting events at species margins but instead found that while mast years are indeed less frequent, the interannual variation was lower toward the margins. The observed patterns suggest that populations growing at the margins may invest more resources in low seed production years compared with their conspecifics, hedging their bets in these more challenging environments.

## INTRODUCTION

For many long‐lived plant species from diverse taxa and ecosystems, seed production is highly variable between years, and individuals within populations synchronize their reproductive effort (Dale et al., 2021; Pearse et al., 2016). The resulting population‐level variability of seed production is known as masting (Kelly, 1994; Pearse et al., 2016). A widely held hypothesis to explain masting variability is that plants growing in resource‐poor, stressful, or marginal environments show more pronounced masting (the “environmental stress,”, or “productivity gradient” hypothesis) (Allen et al., 2012; Kelly & Sork, 2002; Pearse et al., 2017; Roland et al., 2014; Satake & Bjørnstad, 2008; Wion et al., 2020, 2023). Here, we show that masting varies over environmental gradients, but that the environmental stress hypothesis does not explain this spatial variation.

We require a thorough understanding of what drives within‐species variation in masting, given the important effects of masting on ecosystems. Masting increases reproductive efficiency through economies of scale that decrease seed predation rates and improve pollination efficiency (Kelly & Sork, 2002; Pearse et al., 2016). Understanding these reproductive patterns in plants is increasingly important in the context of climate change, because plant reproduction is a critical component of forest dynamics following climate‐driven disturbance and environmental change (Sharma et al., 2022). Climate‐driven changes to mast seeding can severely limit the viability of seeds (Bogdziewicz et al., 2023). Moreover, seeds represent a foundational food source, and masting affects animal populations throughout food webs (Clark et al., 2019).

The environmental stress hypothesis predicts that comparatively more stressful, lower productivity environments have less frequent large seed crops, resulting in higher interannual variability of seed production when compared with less limited populations (Allen et al., 2012; Kelly & Sork, 2002; Pearse et al., 2017; Satake & Bjørnstad, 2008). According to the hypothesis, such environments limit how quickly plants can accumulate sufficient resources to produce a bumper crop. With increasingly challenging growing conditions, we thus expect lower seed production in years following high seed crops (i.e., stronger resource depletion), causing the temporal autocorrelation at lag one year to become more negative (Koenig & Knops, 2000; Sork et al., 1993).

Until now, the environmental stress hypothesis has not been tested at a large scale. Testing is complicated by the fact that many factors can reduce plant productivity, and the stresses experienced by plants vary by species and location. This may help explain why the few studies that have examined the effects of different climate and productivity gradients on masting find contradicting responses, that is, positive, negative, or neutral responses (Table 1). We propose a way to find commonalities between species in their responses to stress gradients by using the concept of “climate marginality.”

**TABLE 1:** Relationships between environmental gradients and metrics capturing temporal reproductive patterns per tree species reported in the literature.

			Direction of effect		
Metric	Gradient	Decrease	Neutral	Increase	Species
CVp	Elevation	Masaki et al. (2020), Roland et al. (2014)			*Fagus crenata, Swida controversa; Picea glauca*
			Lázaro et al. (2006), Masaki et al. (2020), Mencuccini et al. (1995), Mooney et al. (2011)		*Buxus balearica; Quercus serrata, Castanea crenata, Prunus grayana; Picea abies; Pinus ponderosa*
				Buechling et al. (2016), Kelly et al. (2001), Kelly et al. (2008), Masaki et al. (2020), Sullivan and Kelly (2000), Webb and Kelly (1993)	*Picea engelmannii; Nothofagus solandri; Chionochloa pallens, Chionochloa macra; Quercus crispula; Chionochloa rubra*; Subalpine plants
	Elevation & latitude		LaMontagne et al. (2021)		*Abies, Picea, Pinus, Tsuga*
	Latitude		Pearse et al. (2020)		Global scale
	Nitrogen	Tanentzap et al. (2012), Smaill et al. (2011)			*Chionochloa* spp.; *Nothofagus solandri*
	Water availability	Espelta et al. (2008), Wion et al. (2020)			*Quercus humilis; Pinus edulis*
			Bogdziewicz et al. (2020), Espelta et al. (2008), Fernández‐Martínez et al. (2012), Le Roncé (2021)		*Quercus ilex, Phillyrea latifolia, Arbutus unedo; Quercus ilex; Quercus ilex, Quercus pubescens; Quercus ilex*
CVi & S	Water availability		Wion et al. (2023)		*Pinus ponderosa*
AR(1)	Water availability	Barringer et al. (2013)			*Quercus lobata, Quercus agrifolia*
Periodicity	Elevation	Allen et al. (2012)			*Nothofagus solandri*
Mast frequency	Elevation	Mencuccini et al. (1995)			*Picea abies*
	Nitrogen			Tanentzap et al. (2012)	*Chionochloa* spp.

*Note*: Reference details in Appendix S1: Section S1.

Abbreviations: AR(1), 1‐year lagged autocorrelation; CVi: coefficient of variation of tree‐level seed crop size; CVp, coefficient of variation of population‐level seed crop size; S, between‐tree synchrony.

The limit of a species' range is a manifestation of its ecological niche (Sexton et al., 2009). If the species range edges are near equilibrium (i.e., the species can be found throughout most of its potential distribution), climate marginality theory predicts that populations at the climatic periphery more frequently experience the effect of biotic and abiotic limiting factors compared with populations living in core conditions and that demographic performance would consequently be impaired (Kunstler et al., 2021; Pironon et al., 2017; Sexton et al., 2009). Changes in reproduction patterns toward climatically marginal sites could result from the limited availability of resources, or from higher demands on these resources for maintenance or overcoming increased competition (Gaston, 2009; Roland et al., 2014), or because of more frequent extreme climate events that affect reproduction such as late spring frosts and summer droughts (Journé et al., 2021; Nussbaumer et al., 2020; Willi & Van Buskirk, 2022). All in all, we expect to observe changes in masting behavior across climate marginality gradients, with masting becoming stronger with increasing climate marginality.

Here, we present the first attempt to generalize, across 19 tree species, the effect of environmental stress on masting. While intraspecific variation in masting by populations is widely acknowledged (Crone et al., 2011; Greene & Johnson, 2004; Herrera et al., 1998; Satake & Bjørnstad, 2008), it has remained largely unclear how much intraspecific variation of masting exists within species across space, and what drives this variability. Thus far, studies designed to assess the effect of environmental stress on intraspecific masting variability have been constrained in terms of spatial and species coverage. Now, due to a recent synthesis of data on reproductive behavior in plants (Hacket‐Pain et al., 2022), we are able to test the long‐standing environmental stress hypothesis across species and extensive environmental gradients.

We expect to observe parabolic relationships between climate variables and masting metrics. Specifically, we expect (1) seed production to be more variable, (2) more negatively autocorrelated through time (indicative of a stronger resource limitation; Koenig et al., 2003), and (3) have less frequent large seed crops in populations growing in marginal climates. In contrast, range centers should be characterized by less variable, less autocorrelated, and more frequent large seed crops.

## MATERIALS AND METHODS

We describe the variability of masting for populations from 19 tree species occurring in mid‐to‐high latitudes using three masting metrics. Subsequently, we test whether these metrics vary along climate marginality gradients in the ways predicted by the environmental stress hypothesis. For the six best‐represented species in terms of data coverage, we also fit models to gain insight into the species‐specific responses behind the overall, across species, pattern. All models were fitted in R v. 4.3.1 (R Core Team, 2023).

### Intraspecific variation in reproductive patterns

#### Reproduction time series

We obtained time series of annual reproduction from the open‐access MASTREE+ database (Hacket‐Pain et al., 2022). This database collates geo‐referenced reproductive time series from perennial plant species across the world. We subset MASTREE+ to seed, fruit, and cone production time series of species which had extensive temporal (≥10 years of observation) and spatial (≥10 sites) replication. From here on, seeds, fruit, and cones will be referred to as seeds, for simplicity. *Quercus robur* and *Quercus petraea* time series were grouped together, as the often sympatric species have high hybridization rates (Abadie et al., 2012), and synchronize their seed production (Bogdziewicz et al., 2017). We only included series representing stand and patch scale populations (excluding [super‐]regional scale records) with high‐precision spatial references, to accurately link reproductive behavior and climate. Moreover, to ensure the masting metrics could be meaningfully calculated and compared, we excluded series which lacked a unit (i.e., ordinal data, index data) or which had synchrony‐based units (i.e., “% of individuals reproducing”). Comparable collection methods were grouped together before conducting the analyses, and classified as either seed trap data, seed counts, or harvest records. This approach resulted in a dataset of 8082 annual records, forming 437 time series of the reproductive behavior of 19 plant species in 357 locations (Appendix S1: Table S1). On average, time series were 18.5 years long (median: 14, range: 10–62).

#### Temporal reproductive patterns

Three metrics capturing temporal reproductive patterns were calculated for each time series to describe masting variability. Firstly, we calculated the coefficient of variation (CVp), the standard deviation divided by the mean annual seed crop size (i.e., number of seeds). Temporal autocorrelation was calculated with the 1‐year lagged autocorrelation, AR(1), and obtained with the Acf function in R (Hyndman et al., 2022). Lastly, we calculated the proportion of high seed crop years, Psd. High seed crop years (“mast years”) were defined as years when the standardized annual deviate of reproductive effort exceeded the absolute magnitude of the largest deviate below the mean, as proposed by LaMontagne and Boutin (2007). Additionally, we calculated the recently introduced kCVp, a bounded alternative to CVp, which was found to increase statistical power when compared with CVp when testing for latitudinal patterns in masting (Lobry et al., 2023). This metric can be calculated from the CVp by dividing the squared CVp by 1 + the squared CVp and subsequently taking the square root.

### Environmental gradients

#### Climate data and species' ranges

Climate data for each location were extracted from WorldClim v. 2.1 (Fick & Hijmans, 2017). Temperature variables were adjusted (lapse rate: 0.65°C/100 m) when the elevation of MASTREE+ time series was known and deviated from the SRTM data used for WorldClim (Fick & Hijmans, 2017). To assess the climate gradients covered by our sampling, we extracted climate data for species distributions. Species distributions for all but two species were obtained from EUFORGEN (2022) and the Atlas of United States Trees (Petry, 2021). The range of *Fagus crenata* was digitized from Kobashi et al. (2006), and a digitized range of *Araucaria araucana* was provided by CONICET‐UNS (M. Hadad, CONICET‐UNS, unpublished data, 2023). For most species in the dataset, wide spatial and elevational gradients were sampled (Appendix S1: Figure S1 and Table S1), capturing a large part of the climatic envelope (Appendix S1: Figure S2 and Table S5).

#### Linear models

##### Across‐species patterns

We tested for intraspecific effects of climate gradients on temporal reproductive patterns (i.e., CVp, AR1, and Psd) using the glmmTMB package (v. 1.1.7; Brooks et al., 2017). To allow for comparisons between CVp and kCVp, we also substituted kCVp in the final CVp models.

While the inclusion of the species as a predictor captures between‐species differences in the dependent variable, this does not account for potential between‐species differences in the other predictors (Pearse et al., 2020; Van de Pol & Wright, 2009). We therefore subject‐centered all dependent and independent numeric variables by subtracting the species' (i.e., “subject”) mean of each respective variable, except for climate variables (N.B. models using these data are referred to as “subject‐centered models”). For climate variables, we subtracted the median value observed in the species' range, which allowed for explicit testing of our hypotheses, as the centered value captures distance from the core climate.

For each metric, we ran two models, namely (1) an annual average model, including mean annual temperature (MAT), annual precipitation (AP), the interaction between MAT and AP, as well the second order polynomials of MAT and AP as predictors, and (2) a seasonal model, using the temperatures and precipitation of the coldest (cq) and hottest (hq) quarter of the year, the temperature–precipitation interactions for both quarters (i.e., $T_{\mathrm{cq}}\times P_{\mathrm{cq}},T_{\mathrm{hq}}\times P_{\mathrm{hq}}$) and the second order polynomials of both temperature and precipitation variables. Since all sites are situated in mid‐to‐high latitudes, the hottest and coldest quarter correspond to summer and winter, respectively. We accounted for methodological differences and time series properties by including as predictors (1) time series length, (2) a categorical classification of the measured reproductive variable (i.e., “cone,” “fruit,” “seed”), and (3) the data collection method (i.e., “harvest record,” “seed trap,” “count‐based method”). Since centering removes the average effect of species, no random factor was used to capture between‐species differences. The AR(1) models showed a statistically significant latitudinal trend in the model residuals, and we therefore included subject‐centered “Latitude” as a predictor in these models. All numeric predictor variables were scaled using the root mean square to ensure model convergence. We allowed the dispersion of residuals to vary as a function of species, collection method, and reproductive variable. Models were examined and validated with DHARMa (v. 0.4.6), by simulating model residuals and plotting these against observations and predictors to check for misfits, as well as testing for correct distribution (KS test), dispersion, and outliers (Hartig & Lohse, 2022). Additionally, we tested for collinearity with car (v. 3.1.2; Fox & Weisberg, 2019). To assess the significance of the linear terms in these across‐species models, we conducted likelihood ratio tests. Standard *p*‐values reflect conditional hypotheses and are not suitable for evaluating the independent contributions of predictors (*x*) due to the inclusion of quadratic terms (*I*(*x* ^2^)). Specifically, for each linear climate predictor, we compared a “full” model—the model described above but excluding the quadratic and interaction terms involving the focal predictor—to a “reduced” model in which the linear term itself was also removed (see Appendix S1: Table S2).

##### Species‐specific patterns

Since mean trends may obscure ecologically relevant processes, we also fitted species‐specific linear models with glmmTMB for CVp, AR(1), and Psd for six species with ≥30 observations. We used the same predictors as those used in the subject‐centered models, but since only a single species was examined in each model, variables other than the climate variables were not species‐centered. Variable scaling was not required for convergence. If there was no variation in reproductive variable or collection method for a species, the variable could not be included in the model. The collection method predictor was removed from the *Picea glauca* models because it exceeded the collinearity threshold ($\mathrm{GVI}F^{\frac{1}{2\times \mathrm{df}}}>\sqrt{10}$).

## RESULTS

### Intraspecific variation

**FIGURE 1:**
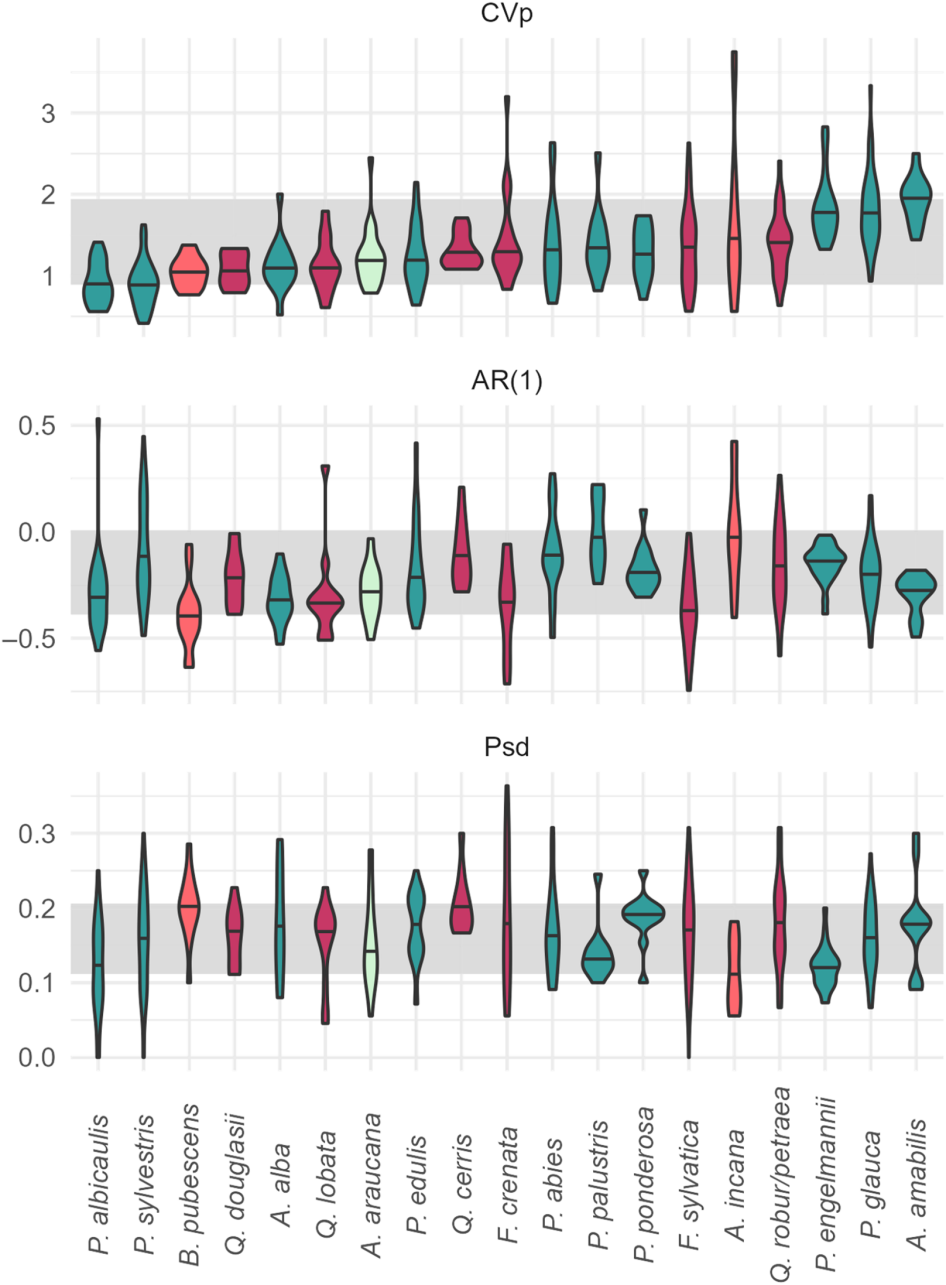
Intraspecific variation in masting metrics often exceeds interspecific variation. The shaded gray rectangle indicates the interspecific range in metric values. The colors of the violin plots indicate plant families, with blue‐greens reserved for gymnosperms and orange‐reds for angiosperms. AR(1), 1‐year lagged autocorrelation; CVp, coefficient of variation of population‐level seed crop size; Psd, the proportion of large seed production years. See Figure 2 for genus names.

**FIGURE 2:**
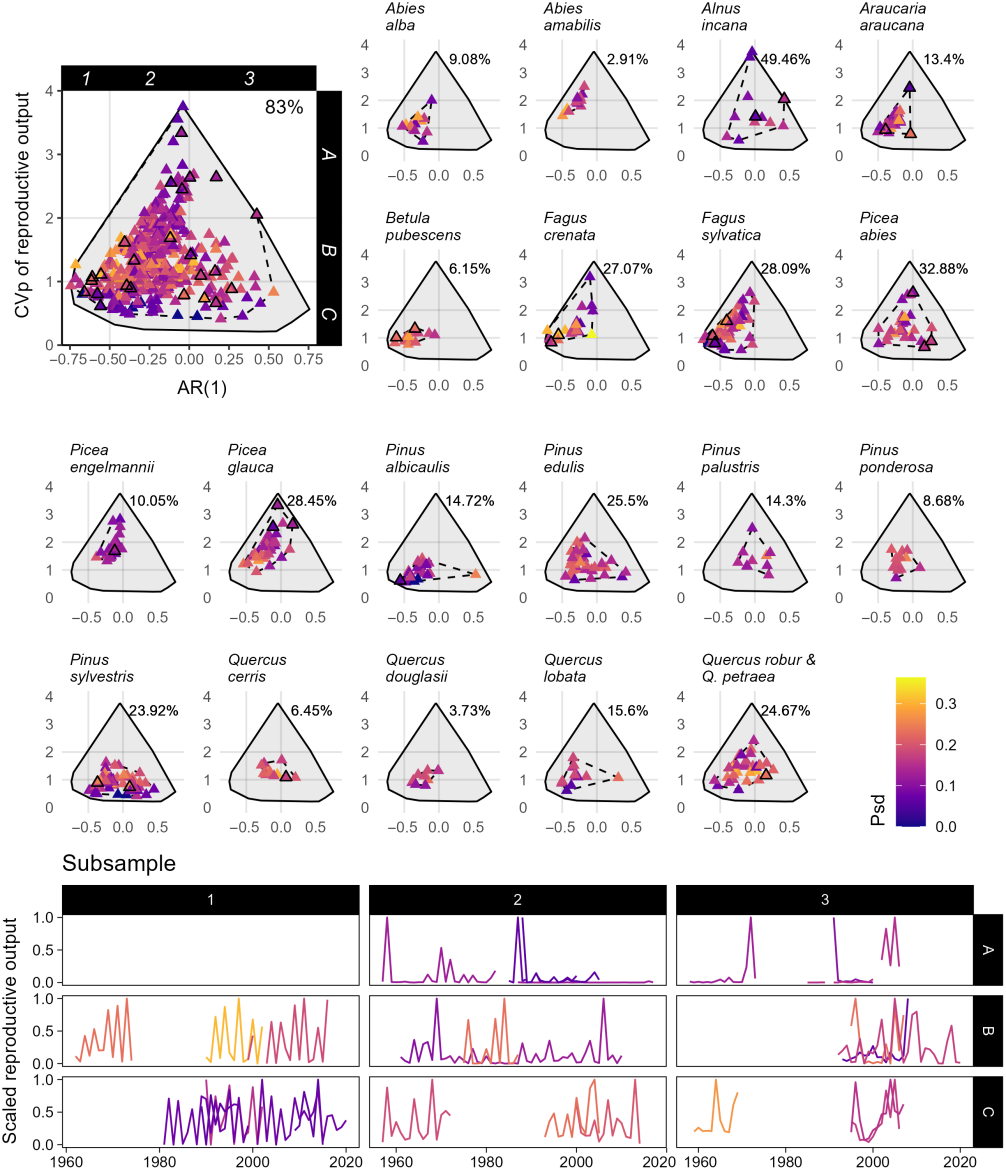
Within‐species variation in masting metrics covers a large part of across‐species masting metric space for some, but not all tree species. The top‐left plot shows how three metrics co‐vary. (AR(1), 1‐year lagged autocorrelation; CVp, coefficient of variation of population‐level seed crop size; Psd, the proportion of large seed production years.) Dashed‐line convex hull: Variation of CVp and AR(1) across all analyzed species. Solid‐line convex hull: the CVp and AR(1) metric space of all 378 species (1018 time series) in MASTREE+ with time series matching sub‐setting criteria, irrespective of whether they had adequate spatial replication for inclusion in our analyses. Species‐specific metric space is shown in surrounding plots. Percentages in the top‐right corner of plots show the ratio of the solid to dashed convex hull area. Outlined time series in the top‐left plot are plotted in the “Subsample” plots (3 random time series [1960–2020] per region; regions marked with 1–3 and A–C) to illustrate example time series. Example time series are scaled by their maximum value.

Reproductive patterns varied widely within species (Appendix S1: Table S1; Figures 1 and 2). Notably, for 14 out of 19 species, the observed intraspecific range of CVp values was greater than the interspecific variation in mean CVp (0.89–1.94, Appendix S1: Table S1; Figure 1). Similarly, the intraspecific variation in AR(1) of 16 species exceeded the observed interspecific variation in mean AR(1) (−0.39–0.01, Appendix S1: Table S1; Figure 1). The majority of time series, both within and across species, had negative AR(1) values. All intraspecific ranges in Psd exceeded the interspecific range in mean Psd (0.11–0.21, Appendix S1: Table S1; Figure 1).

The species‐specific metric space plots in Figure 2 highlight how intraspecific variation in CVp and AR(1) in some species covers large portions of the across‐species metric space (e.g., *Alnus incana*: 49.5%, *Picea abies*: 32.9%). In contrast, some species have high intraspecific variability in only one metric (e.g., CVp of *Picea engelmannii*, AR(1) of *Pinus sylvestris*), or relatively low intraspecific variability in both CVp and AR(1) (e.g., *Abies amabilis*: 2.9%, *Quercus douglasii*: 3.7%).

### Climate marginality

#### Across‐species patterns

**FIGURE 3:**
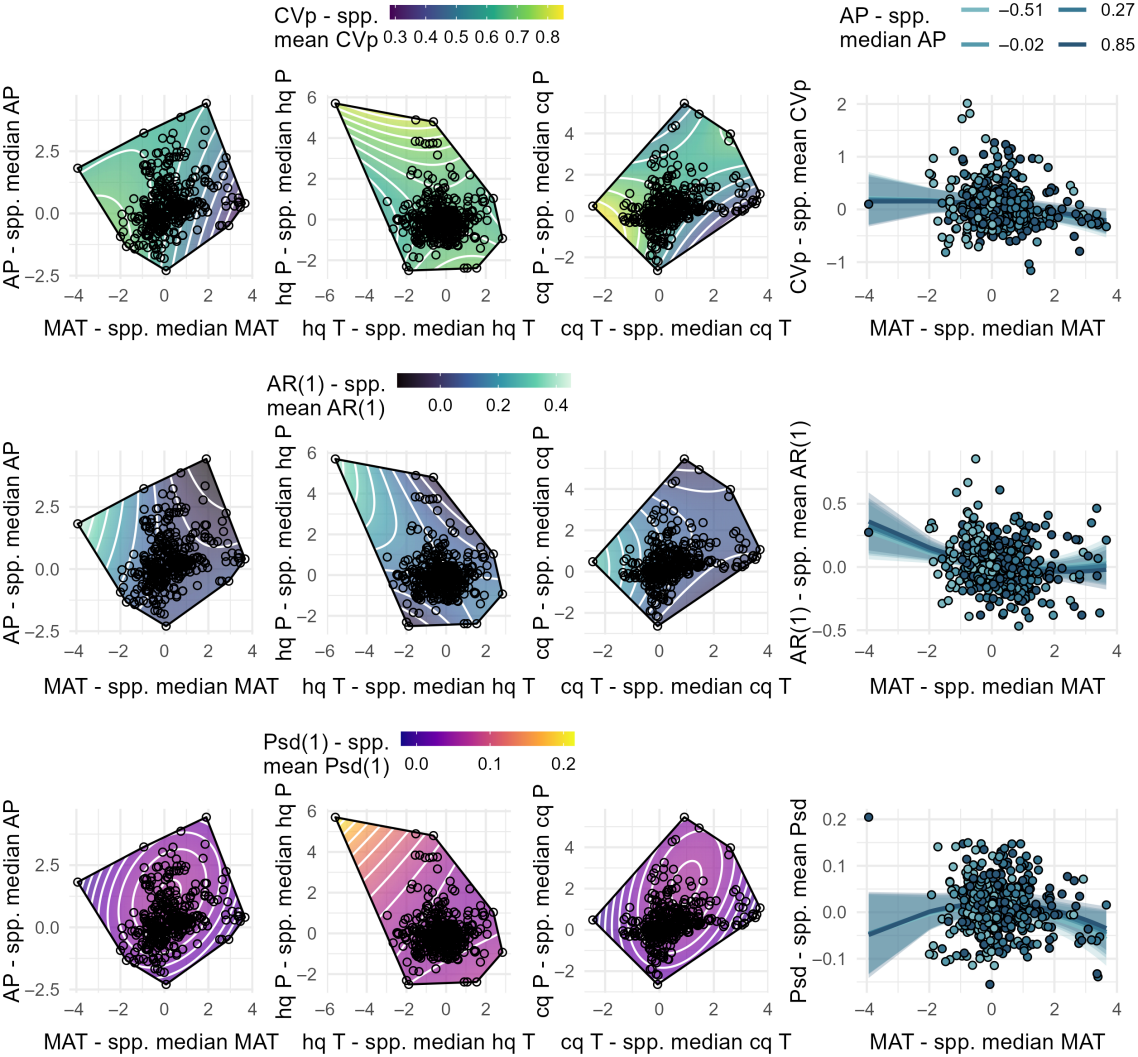
Climate marginality explains variation in masting, but patterns diverge from predictions under the environmental stress hypothesis. The three columns on the left show response surfaces for the subject‐centered models of masting metrics CVp, AR(1), and Psd (AR(1), 1‐year lagged autocorrelation; CVp, coefficient of variation of population‐level seed crop size; Psd, the proportion of large seed production years) at baseline levels of the centered and scaled predictor variables. The annual average model is found in the first column, and the results of the seasonal model (cq, coldest quarter; hq, hottest quarter; P, precipitation; T, temperature) are found in columns 2–3. The boundaries of the response surfaces in columns 1–3 were determined by a convex hull of climate data of the time series (open circles). The right‐most column shows an alternative representation of the annual model found in the first column. Specifically, it shows the marginal effects (and associated 95% CIs, shaded) for mean annual temperature (MAT) at different levels of annual precipitation (AP; blue colors) in the annual CVp, AR(1), and Psd models, alongside partial residuals. Spp., species.

Large seeding years were less frequent toward more marginal climates, which is consistent with predictions of the environmental stress hypothesis. In the annual model, Psd responded to within‐species change in ${\mathrm{MAT}}^{2}$ (Figure 3, climate effects: Table 2, full model: Appendix S1: Table S3). Sites with a climate closely matching the species' range median MAT displayed more frequent masting. It should be noted that while the concave relationship between Psd and AP best fitted the observations, this relationship was not statistically significant. Squared winter temperatures predicted Psd in the seasonal model, with the lowest masting frequencies occurring at cold and warm sites. Thus, annual climate patterns appear to be more closely related to climatic conditions in the winter rather than in the summer.

**TABLE 2:** Relationships between climate variables and masting metrics CVp, AR(1), and Psd found using subject‐centered annual average and seasonal models.

	CVp			AR(1)			Psd		
Term	Estimate	*z*	*p*‐value	Estimate	*z*	*p*‐value	Estimate	*z*	*p*‐value
Annual									
AP	9.73 × 10^−3^ (3.07 × 10^−2^)	0.32	0.75	1.49 × 10^−3^ (1.29 × 10^−2^)	0.12	0.91	**9.67 × 10** ^**−3**^ **(4.51 × 10** ^**−3**^ **)**	**2.14**	**0.03**
AP^2^	−1.40 × 10^−3^ (1.38 × 10^−2^)	−0.10	0.92	−2.86 × 10^−3^ (5.32 × 10^−3^)	−0.54	0.59	−3.75 × 10^−3^ (2.03 × 10^−3^)	−1.84	0.07
MAT	**−6.21 × 10** ^**−2**^ **(2.69 × 10** ^**−2**^ **)**	**−2.31**	**0.02**	**−3.45 × 10** ^**−2**^ **(1.10 × 10** ^**−2**^ **)**	**−3.13**	**<0.01**	−1.31 × 10^−3^ (4.08 × 10^−3^)	−0.32	0.75
MAT × AP	1.43 × 10^−2^ (1.95 × 10^−2^)	0.73	0.47	−1.51 × 10^−2^ (9.75 × 10^−3^)	−1.55	0.12	2.37 × 10^−3^ (3.55 × 10^−3^)	0.67	0.50
MAT^2^	−8.66 × 10^−3^ (8.77 × 10^−3^)	−0.99	0.32	9.95 × 10^−3^ (5.95 × 10^−3^)	1.67	0.09	**−4.23 × 10** ^**−3**^ **(2.06 × 10** ^**−3**^ **)**	**−2.05**	**0.04**
Seasonal									
hq P	−4.08 × 10^−3^ (2.77 × 10^−2^)	−0.15	0.88	2.29 × 10^−3^ (1.09 × 10^−2^)	0.21	0.83	−1.74 × 10^−3^ (3.81 × 10^−3^)	−0.46	0.65
hq P^2^	6.14 × 10^−3^ (1.22 × 10^−2^)	0.50	0.61	**−1.14 × 10** ^**−2**^ **(3.98 × 10** ^**−3**^ **)**	**−2.87**	**<0.01**	1.35 × 10^−3^ (1.60 × 10^−3^)	0.84	0.40
hq T	5.15 × 10^−3^ (2.44 × 10^−2^)	0.21	0.83	2.32 × 10^−4^ (8.66 × 10^−3^)	0.03	0.98	−1.07 × 10^−3^ (3.21 × 10^−3^)	−0.33	0.74
hq T × hq P	−4.49 × 10^−3^ (1.83 × 10^−2^)	−0.25	0.81	**−2.18 × 10** ^**−2**^ **(7.17 × 10** ^**−3**^ **)**	**−3.05**	**<0.01**	−2.91 × 10^−3^ (2.70 × 10^−3^)	−1.08	0.28
hq T^2^	−5.03 × 10^−3^ (1.33 × 10^−2^)	−0.38	0.70	−1.79 × 10^−3^ (5.07 × 10^−3^)	−0.35	0.72	1.07 × 10^−3^ (1.91 × 10^−3^)	0.56	0.58
cq P	1.96 × 10^−2^ (3.15 × 10^−2^)	0.62	0.54	1.78 × 10^−2^ (1.25 × 10^−2^)	1.43	0.15	6.74 × 10^−3^ (4.87 × 10^−3^)	1.39	0.17
cq P^2^	−1.36 × 10^−2^ (1.01 × 10^−2^)	−1.35	0.18	**−8.32 × 10** ^**−3**^ **(3.79 × 10** ^**−3**^ **)**	**−2.20**	**0.03**	−2.23 × 10^−3^ (1.53 × 10^−3^)	−1.46	0.14
cq T	**−1.02 × 10** ^**−1**^ **(4.45 × 10** ^**−2**^ **)**	**−2.30**	**0.02**	**−5.96 × 10** ^**−2**^ **(1.92 × 10** ^**−2**^ **)**	**−3.10**	**<0.01**	8.26 × 10^−3^ (5.98 × 10^−3^)	1.38	0.17
cq T × cq P	**4.46 × 10** ^**−2**^ **(2.11 × 10** ^**−2**^ **)**	**2.11**	**0.03**	8.31 × 10^−3^ (1.40 × 10^−2^)	0.59	0.55	3.42 × 10^−3^ (4.53 × 10^−3^)	0.75	0.45
cq T^2^	−4.68 × 10^−3^ (1.45 × 10^−2^)	−0.32	0.75	7.67 × 10^−3^ (8.76 × 10^−3^)	0.88	0.38	**−8.13 × 10** ^**−3**^ **(2.53 × 10** ^**−3**^ **)**	**−3.21**	**<0.01**

*Note*: SEs within parentheses. *p*‐values were calculated with Wald tests via the summary() function. For linear predictors, these *p*‐values reflect conditional hypotheses and should not be interpreted as evidence of independent contributions due to the inclusion of quadratic terms as *I*(*x* ^2^). Significant effects are in bold, and underlined values indicate that the independent linear predictor was also significant in likelihood ratio tests comparing a model with the predictor (excluding interactions and quadratic terms) to one without it, to test the independent contributions. These likelihood ratio results can be found in Appendix S1: Table S2.

Abbreviations: AP, annual precipitation; AR(1), 1‐year lagged autocorrelation; cq, coldest quarter; CVp, coefficient of variation of population‐level seed crop size; hq, hottest quarter; MAT, mean annual temperature; P, precipitation; Psd, the proportion of large seed production years; T, temperature.

Yet, these changes in the frequency of large seeding years did not result in a higher CVp toward the margins, as predicted by the environmental stress hypothesis. CVp varied significantly with temperature, with the annual model predicting lower CVp in the warmest sites, and no effect of precipitation was observed (Figure 3, climate effects: Table 2, full model: Appendix S1: Table S3). The seasonal model shows that the effect of winter temperature depends on winter precipitation. For populations growing in sites close to the species' median winter precipitation level, the CVp declined with increasing winter temperatures. Combined, these findings show that the relationships between CVp and marginality gradients are more complex than anticipated under the environmental stress hypothesis. The simulated kCVp model residuals showed deviations from normality (Kolmogorov–Smirnov; annual model: *D* = 0.08, *p* < 0.05; seasonal model: *D* = 0.09, *p* < 0.01), but the model results are presented in Appendix S1: Figure S3 and Table S3 to allow for comparisons with the CVp models. The results from the annual average kCVp model imply that year‐to‐year variability in seed production decreases toward cold and warm margins, not just toward the warmer sites as in the CVp model. These findings directly contrast with the predictions under the environmental stress hypothesis. While the detected climate patterns differ from the annual average CVp model, the same methodological variables were found to have effects in both the annual average CVp and kCVp models (i.e., reproductive variable and data collection method). In the seasonal model, a similarly nuanced story emerges, and the observed relationships between climate and the temporal variability of seed production also differed. A concave relationship was found between squared winter temperatures and kCVp, rather than the significant interaction between winter temperature and precipitation detected in the CVp model. Both models show that kCVp is not simply a more powerful variant of CVp. Instead, different relationships may be detected.

Lastly, how AR(1) varies along climate marginality gradients is not well explained by the predictions under the environmental stress hypothesis. Intraspecific variation in AR(1) varied with MAT in the annual climate model, where sites with higher MATs had lower AR(1) values (Figure 3, climate effects: Table 2, full model: Appendix S1: Table S3). Similarly to the annual model, the seasonal model indicates AR(1) was lower when sites experienced warmer temperatures in winter. AR(1) decreased toward both winter precipitation margins, that is, toward dry and wet sites. The effect of the summer temperatures depended on precipitation; lower AR(1) occurred in drier and colder sites, as well as in warmer and wetter sites. Summer precipitation also showed a parabolic relationship with AR(1), where AR(1) was lowest at the wettest and driest sites. Thus, we only found limited evidence for concave relationships in the seasonal model.

Overall, we find that masting patterns vary across marginality gradients. However, this variation differs from predictions under the environmental stress hypothesis (Table 3). In addition to these diverging relationships, we also find that there is substantial, and potentially ecologically meaningful, variation around these predictions (see partial residuals, Figure 3). This indicates that factors other than climate marginality or methodological differences play an important role in driving masting variability.

**TABLE 3:** Summary of main findings, showing whether the relationship between climate and masting metrics matched predictions under the environmental stress hypothesis.

	Environmental stress hypothesis			
Metric	Prediction	Climate variable	Supported	Results
Psd	Less frequent masting in more marginal climates	MAT	Yes	Less frequent masting in cool and warm sites
		AP	Partially	Less frequent masting in dry and wet sites, but not significant
AR(1)	More negative autocorrelation in more marginal climates	MAT	No	More negative autocorrelation in warm sites
		AP	No	No significant effects
CVp	Higher variability in more marginal climates	MAT	No	Lower variability in warm sites
		AP	No	No significant effects
kCVp	Higher variability in more marginal climates	MAT	No	Lower variability in cold & warm sites
		AP	No	No significant effects

Abbreviations: AP, annual precipitation; AR(1), 1‐year lagged autocorrelation; CVp, coefficient of variation of population‐level seed crop size; MAT, mean annual temperature; Psd, the proportion of large seed production years.

#### Species‐specific patterns

There is always a risk that ecologically meaningful relationships go undetected if trends are only tested at a general level. Or, phrased differently, there is a chance that the hypothesis holds, but only for a subset of species. Consequently, we tested how masting metrics varied across annual climate marginality gradients for the six best‐sampled species (Appendix S1: Table S4). Here, we reveal, where applicable, evidence of species‐specific responses matching the predictions of the environmental stress hypothesis.

Evidence in line with our prediction that Psd would be lower in the climatic margins was found for *P. abies* and *P. sylvestris*. Specifically, we observed concave effects on Psd of MAT for *P. abies* and of AP for *P. sylvestris*, although highest Psd values were found in sites with higher‐than‐median precipitation in this species.

The relationship between CVp and climate did not generally match the predicted convex pattern of highest variability in more marginal climates, with the exceptions of the effect of MAT on *F. sylvatica* and the effect of AP on *Pinus edulis* (although this later effect was not statistically significant [*p* = 0.06]).

For AR(1), the evidence in line with predictions is the concave relationship between AR(1) and AP in *Q. robur/petraea*, and between AP and temperature and AR(1) in *P. abies*.

It is worth noting that these species‐specific models have relatively small sample sizes (Appendix S1: Table S1), and therefore have lower power than the subject‐centered models. Yet, these models reveal that species responses are diverse.

## DISCUSSION

The environmental stress hypothesis fails to explain intraspecific masting variation when evaluated across climate marginality gradients (Table 3). Under the environmental stress hypothesis, we would predict higher interannual variation and, simultaneously, less frequent large seeding events at species' margins (Allen et al., 2012; Kelly & Sork, 2002; Pearse et al., 2017; Roland et al., 2014; Satake & Bjørnstad, 2008; Wion et al., 2020, 2023). Instead, we found that while high seeding years indeed became less frequent toward the margins, the interannual variation becomes lower instead of higher toward the warm margin (or toward warm and cold margins for the alternative kCVp metric). Combined, these patterns signal more constant seed production toward the margins, not stronger masting. The patterns in the temporal autocorrelation are also indicative of more constant seed production. When there are few large seeding events (which happens toward the warm margins), this average relationship is determined mostly by pairs of subsequent low reproduction years. If populations shift from zero or extremely low seed output in low seed crop years to increased seed production in some of these years, the AR(1) would decrease, but the overall seed crop would become more constant. Overall, our results provide solid support that masting changes across climate gradients, but not in the way expected under the environmental stress hypothesis.

Our work helps to develop a clearer picture of how reproduction varies across climate space. Various studies have found that the number of flowers, fruits, and seeds produced at the edge of the geographic distribution range was comparable to that produced at the geographic center (Abeli et al., 2014; Pironon et al., 2017). However, in masting populations, reproductive outcomes depend not only on the number of seeds produced but also on the temporal variability of these seed crops (Bogdziewicz et al., 2023). The more constant reproduction observed toward climate margins has many potential consequences for ecosystem dynamics. For example, less frequent mast years of large‐seeded species like beech and oak lead to declines in seed consumer populations (Touzot et al., 2020). In addition to the general, across species, trends in masting variability across space, we find evidence that species‐specific responses to climate gradients are diverse. This implies that the masting of some species is more responsive to climatic variation than others.

In this analysis, the spatial sampling was limited by the availability of long‐term reproductive data. Future efforts which deliberately sample marginal populations over decades would be highly valuable, and would help to detect species‐specific signals of stress on masting. However, such data are currently still limited. Regardless, our data were sampled across strong gradients, which, for the best‐sampled species, cover the reported climatic envelope of the species (see Appendix S1: Section S2).

Our first large‐scale assessment of within‐species variation in masting shows that it can exceed the among‐species variation. *P. glauca*, for example, is a species that is typically associated with masting (i.e., high variability of seed production). Yet, we found that the CVp of populations of this species ranged from very high (3.34) to moderately low (0.93). This range is more than double the among‐species range in mean CVp (0.89–1.94). Such high intraspecific variability provides further evidence of the pitfalls of classifying species as “masting” or “non‐masting” (Kelly, 2023), and illuminates the need for caution when working with species‐level averages: such models implicitly assume that species means are meaningful and that the intraspecific variation in the trait of interest is lower than the interspecific variation (Garamszegi, 2014; Westerband et al., 2021). Moreover, the striking extent of intraspecific variation in masting suggests that the downstream consequences of masting may be more spatially variable than assumed. Given the important effects of masting on wider ecosystems, it is crucial that we develop a thorough understanding of what causes masting to vary so much between populations.

Masting at species climate margins was characterized by less frequent large seeding years and lower interannual variation in seed production. The observed patterns might be explained by larger reproductive investment in small seed production years toward species margins, as has been observed for European beech (Müller‐Haubold et al., 2015). Theory predicts that the costs of masting, such as the risk of dying before producing bumper crops, increase with decreased adult survivorship and that longer periods between large seeding years elevate these costs (Journé et al., 2023; Waller, 1979). Therefore, lower survival rates at marginal populations together with lower frequencies of producing large seeding years may translate into higher reproductive investment in low seed production years, a form of bet hedging in these more challenging environments. Moreover, lower benefits to masting may also occur in these sites if seed predator pressures are lower (Alexander et al., 2007; Vaupel & Matthies, 2012). Future work considering masting variability alongside factors determining the cost–balance equation of masting benefits is therefore required.

The observed variance in masting patterns around the effects of climate marginality suggests that factors beyond climate contribute to intraspecific masting variability. The importance of local environmental effects was demonstrated in *Pinus ponderosa*, where intraspecific variation in masting was associated with stand characteristics including stand density, tree size, and age (Wion et al., 2023). Plant ontogeny was also found to be an important determinant of masting in some temperate forest trees (Pesendorfer et al., 2020). If these stand characteristics explain a large proportion of variability in masting, then the effects of climate change on masting and the availability of viable seeds at the climatic margins might be alleviated through appropriate management strategies (Bogdziewicz et al., 2023). A limited number of studies indicate that additional reproductive variability is explained by provenance (Caignard et al., 2021; Mark, 1965), but data from reciprocal transplant or common gardens established along climate gradients are still needed.

Due to the rapid accumulation of data on reproductive behaviors (Hacket‐Pain et al., 2022; Pearse et al., 2020), we have been able to test the long‐standing environmental stress hypothesis and unveil the remarkable within‐species variation in masting. We find masting variation across climate space is inconsistent with the environmental stress hypothesis. High seed crop years are less frequent at the climatic periphery, but, importantly, the year‐to‐year variability of seed crops is lower toward the warm margin (or warm and cold margins for the alternative kCVp metric). As climate change may drive more populations to the climatic periphery, it is urgent that we establish if the spatial patterns observed here translate into temporal patterns. Recent work shows that this is not the case in European beech (Foest et al., 2024). Moreover, more work is required to establish whether changes in masting result in lower reproductive efficiency. Lower interannual variation at marginal populations may translate into less effective predator satiation, higher losses to seed predators, and lower reproductive efficiency (Zwolak et al., 2022). The large unexplained variation around the effects of climate not only sets important challenges, but also offers management potential (e.g., managing stand age and structure). If the source of this variability results from stand characteristics, we may be able to develop appropriate management strategies to optimize reproductive efficiency.

## AUTHOR CONTRIBUTIONS

Jessie J. Foest, Andrew Hacket‐Pain, and Thomas Caignard conceptualized the study, and Jessie J. Foest led the development of the methodology, with support from Andrew Hacket‐Pain, Ian S. Pearse, and Thomas Caignard. Investigation, formal analysis, and data visualization were performed by Jessie J. Foest. Jessie J. Foest wrote the original draft, and all authors contributed critical feedback on the manuscript. Andrew Hacket‐Pain provided supervision.

## CONFLICT OF INTEREST STATEMENT

The authors declare no conflicts of interest.

## Supporting information


Appendix S1.

## Data Availability

Data and code (Foest et al., 2025) are available in Figshare at https://doi.org/10.6084/m9.figshare.26355463.v1.

## References

[ecy70076-bib-0001] Abadie, P. , G. Roussel , B. Dencausse , C. Bonnet , E. Bertocchi , J.‐M. Louvet , A. Kremer , and P. Garnier‐Géré . 2012. “Strength, Diversity and Plasticity of Postmating Reproductive Barriers between Two Hybridizing Oak Species (*Quercus robur* L. and *Quercus petraea* (Matt) Liebl.).” Journal of Evolutionary Biology 25: 157–173. 22092648 10.1111/j.1420-9101.2011.02414.x

[ecy70076-bib-0002] Abeli, T. , R. Gentili , A. Mondoni , S. Orsenigo , and G. Rossi . 2014. “Effects of Marginality on Plant Population Performance.” Journal of Biogeography 41: 239–249.

[ecy70076-bib-0003] Alexander, H. M. , S. Price , R. Houser , D. Finch , and M. Tourtellot . 2007. “Is There Reduction in Disease and Pre‐Dispersal Seed Predation at the Border of a Host Plant's Range? Field and Herbarium Studies of *Carex blanda* .” Journal of Ecology 95: 446–457.

[ecy70076-bib-0004] Allen, R. , N. Mason , S. Richardson , and K. Platt . 2012. “Synchronicity, Periodicity and Bimodality in Inter‐Annual Tree Seed Production along an Elevation Gradient.” Oikos 121: 367–376.

[ecy70076-bib-0005] Bogdziewicz, M. , D. Kelly , A. Tanentzap , P. Thomas , J. Foest , J. Lageard , and A. Hacket‐Pain . 2023. “Reproductive Collapse in European Beech Results from Declining Pollination Efficiency in Large Trees.” Global Change Biology 29: 4595–4604. 37177909 10.1111/gcb.16730

[ecy70076-bib-0006] Bogdziewicz, M. , D. Kelly , P. Thomas , J. G. Lageard , and A. Hacket‐Pain . 2020. “Climate Warming Disrupts Mast Seeding and its Fitness Benefits in European Beech.” Nature Plants 6: 88–94. 32042155 10.1038/s41477-020-0592-8

[ecy70076-bib-0007] Bogdziewicz, M. , J. Szymkowiak , I. Kasprzyk , L. Grewling , Z. Borowski , K. Borycka , W. W. Kantorowicz , et al. 2017. “Masting in Wind‐Pollinated Trees: System‐Specific Roles of Weather and Pollination Dynamics in Driving Seed Production.” Ecology 98(10): 2615–2625. 10.1002/ecy.1951. 28722149

[ecy70076-bib-0008] Brooks, M. , K. Kristensen , K. van Benthem , A. Magnusson , C. Berg , A. Nielsen , H. Skaug , M. Mächler , and B. Bolker . 2017. “glmmTMB Balances Speed and Flexibility among Packages for Zero‐Inflated Generalized Linear Mixed Modeling.” The R Journal 9: 378–400.

[ecy70076-bib-0009] Caignard, T. , A. Kremer , X. Bouteiller , J. Parmentier , J.‐M. Louvet , S. Venner , and S. Delzon . 2021. “Counter‐Gradient Variation of Reproductive Effort in a Widely Distributed Temperate Oak (*Quercus petraea*).” Functional Ecology 35: 1745–1755. 36825207 10.1111/1365-2435.13830 PMC7614218

[ecy70076-bib-0010] Clark, J. S. , C. L. Nuñez , and B. Tomasek . 2019. “Foodwebs Based on Unreliable Foundations: Spatiotemporal Masting Merged with Consumer Movement, Storage, and Diet.” Ecological Monographs 89: e01381.

[ecy70076-bib-0011] Crone, E. , E. McIntire , and J. Brodie . 2011. “What Defines Mast Seeding? Spatio‐Temporal Patterns of Cone Production by Whitebark Pine.” Journal of Ecology 99: 438–444.

[ecy70076-bib-0012] Dale, E. , J. Foest , A. Hacket‐Pain , M. Bogdziewicz , and A. J. Tanentzap . 2021. “Macroevolutionary Consequences of Mast Seeding.” Philosophical Transactions of the Royal Society B: Biological Sciences 376: 20200372. 10.1098/rstb.2020.0372 PMC8520783 34657467

[ecy70076-bib-0013] EUFORGEN . 2022. “Species – EUFORGEN European Forest Genetic Resources Programme.” https://www.euforgen.org/species/.

[ecy70076-bib-0014] Fick, S. , and R. Hijmans . 2017. “WorldClim 2: New 1‐km Spatial Resolution Climate Surfaces for Global Land Areas.” International Journal of Climatology 37: 4302–4315.

[ecy70076-bib-0015] Foest, J. J. , M. Bogdziewicz , M. B. Pesendorfer , D. Ascoli , A. Cutini , A. Nussbaumer , A. Verstraeten , et al. 2024. “Widespread Breakdown in Masting in European Beech Due to Rising Summer Temperatures.” Global Change Biology 30: e17307. 38709196 10.1111/gcb.17307

[ecy70076-bib-0016] Foest, J. , T. Caignard , I. Pearse , M. Bogdziewicz , and A. Hacket‐Pain . 2025. “Code and Data: Intraspecific Variation in Masting across Climate Gradients Is Inconsistent with the Environmental Stress Hypothesis.” Figshare. 10.6084/m9.figshare.26355463.v1.40181534

[ecy70076-bib-0017] Fox, J. , and S. Weisberg . 2019. An R Companion to Applied Regression, Third ed. Thousand Oaks, CA: Sage.

[ecy70076-bib-0018] Garamszegi, L. Z. 2014. “Uncertainties Due to Within‐Species Variation in Comparative Studies: Measurement Errors and Statistical Weights.” In Modern Phylogenetic Comparative Methods and Their Application in Evolutionary Biology: Concepts and Practice, edited by L. Z. Garamszegi , 157–199. Berlin, Heidelberg: Springer.

[ecy70076-bib-0019] Gaston, K. 2009. “Geographic Range Limits: Achieving Synthesis.” Proceedings of the Royal Society B: Biological Sciences 276: 1395–1406. 10.1098/rspb.2008.1480 PMC2677218 19324809

[ecy70076-bib-0020] Greene, D. , and E. Johnson . 2004. “Modelling the Temporal Variation in the Seed Production of North American Trees.” Canadian Journal of Forest Research 34: 65–75.

[ecy70076-bib-0021] Hacket‐Pain, A. , J. Foest , I. Pearse , J. LaMontagne , W. Koenig , G. Vacchiano , M. Bogdziewicz , et al. 2022. “MASTREE+: Time‐Series of Plant Reproductive Effort from Six Continents.” Global Change Biology 28(9): 3066–3082. 10.1111/gcb.16130. 35170154 PMC9314730

[ecy70076-bib-0022] Hartig, F. , and L. Lohse . 2022. “DHARMa: Residual Diagnostics for Hierarchical (Multi‐Level/Mixed) Regression Models.”

[ecy70076-bib-0023] Herrera, C. , P. Jordano , J. Guitián , and A. Traveset . 1998. “Annual Variability in Seed Production by Woody Plants and the Masting Concept: Reassessment of Principles and Relationship to Pollination and Seed Dispersal.” American Naturalist 152: 576–594. 10.1086/286191 18811365

[ecy70076-bib-0024] Hyndman, R. , G. Athanasopoulos , C. Bergmeir , G. Caceres , L. Chhay , M. O'Hara‐Wild , F. Petropoulos , S. Razbash , E. Wang , and F. Yasmeen . 2022. “Forecast: Forecasting Functions for Time Series and Linear Models.”

[ecy70076-bib-0025] Journé, V. , T. Caignard , A. Hacket‐Pain , and M. Bogdziewicz . 2021. “Leaf Phenology Correlates with Fruit Production in European Beech (*Fagus sylvatica*) and in Temperate Oaks (*Quercus robur* and *Quercus petraea*).” European Journal of Forest Research 140: 733–744.

[ecy70076-bib-0026] Journé, V. , A. Hacket‐Pain , and M. Bogdziewicz . 2023. “Evolution of Masting in Plants Is Linked to Investment in Low Tissue Mortality.” Nature Communications 14: 7998. 10.1038/s41467-023-43616-1 PMC10693562 38042862

[ecy70076-bib-0027] Kelly, D. 1994. “The Evolutionary Ecology of Mast Seeding.” Trends in Ecology and Evolution 9: 465–470. 21236924 10.1016/0169-5347(94)90310-7

[ecy70076-bib-0028] Kelly, D. 2023. “Mast Seeding: Study of Oak Mechanisms Carries Wider Lessons.” Current Biology 33: R231–R233. 36977386 10.1016/j.cub.2023.02.043

[ecy70076-bib-0030] Kelly, D. , and V. L. Sork . 2002. “Mast Seeding in Perennial Plants: Why, how, where?” Annual Review of Ecology and Systematics 33: 427–447.

[ecy70076-bib-0031] Kobashi, S. , N. Fujii , A. Nojima , and N. Hori . 2006. “Distribution of Chloroplast DNA Haplotypes in the Contact Zone of *Fagus crenata* in the Southwest of Kanto District, Japan.” Journal of Plant Research 119: 265–269. 16583261 10.1007/s10265-006-0271-5

[ecy70076-bib-0032] Koenig, W. , D. Kelly , V. Sork , R. Duncan , J. Elkinton , M. Peltonen , and R. Westfall . 2003. “Dissecting Components of Population‐Level Variation in Seed Production and the Evolution of Masting Behavior.” Oikos 102: 581–591.

[ecy70076-bib-0033] Koenig, W. , and J. Knops . 2000. “Patterns of Annual Seed Production by Northern Hemisphere Trees: A Global Perspective.” American Naturalist 155: 59–69. 10.1086/303302 10657177

[ecy70076-bib-0035] Kunstler, G. , A. Guyennon , S. Ratcliffe , N. Rüger , P. Ruiz‐Benito , D. Z. Childs , J. Dahlgren , et al. 2021. “Demographic Performance of European Tree Species at their Hot and Cold Climatic Edges.” Journal of Ecology 109: 1041–1054.

[ecy70076-bib-0036] LaMontagne, J. , and S. Boutin . 2007. “Local‐Scale Synchrony and Variability in Mast Seed Production Patterns of *Picea glauca* .” Journal of Ecology 95: 991–1000.

[ecy70076-bib-0037] Lobry, J. R. , M.‐C. Bel‐Venner , M. Bogdziewicz , A. Hacket‐Pain , and S. Venner . 2023. “The CV Is Dead, Long Live the CV!” Methods in Ecology and Evolution 14: 2780–2786.

[ecy70076-bib-0038] Mark, A. F. 1965. “Ecotypic Differentiation in Otago Populations of Narrowleaved Snow Tussock, *Chionochloa rigida* .” New Zealand Journal of Botany 3: 277–299.

[ecy70076-bib-0039] Müller‐Haubold, H. , D. Hertel , and C. Leuschner . 2015. “Climatic Drivers of Mast Fruiting in European Beech and Resulting C and N Allocation Shifts.” Ecosystems 18: 1083–1100.

[ecy70076-bib-0040] Nussbaumer, A. , K. Meusburger , M. Schmitt , P. Waldner , R. Gehrig , M. Haeni , A. Rigling , I. Brunner , and A. Thimonier . 2020. “Extreme Summer Heat and Drought Lead to Early Fruit Abortion in European Beech.” Scientific Reports 10: 5334. 32210278 10.1038/s41598-020-62073-0 PMC7093476

[ecy70076-bib-0041] Pearse, I. S. , W. D. Koenig , and D. Kelly . 2016. “Mechanisms of Mast Seeding: Resources, Weather, Cues, and Selection.” New Phytologist 212: 546–562. 27477130 10.1111/nph.14114

[ecy70076-bib-0042] Pearse, I. S. , J. M. LaMontagne , and W. D. Koenig . 2017. “Inter‐Annual Variation in Seed Production Has Increased over Time (1900‐2014).” Proceedings of the Royal Society B: Biological Sciences 284: 1–7. 10.1098/rspb.2017.1666 PMC5740272 29212721

[ecy70076-bib-0043] Pearse, I. S. , J. M. LaMontagne , M. Lordon , A. L. Hipp , and W. D. Koenig . 2020. “Biogeography and Phylogeny of Masting: Do Global Patterns Fit Functional Hypotheses?” New Phytologist 227: 1557–1567. 32315447 10.1111/nph.16617

[ecy70076-bib-0044] Pesendorfer, M. B. , M. Bogdziewicz , J. Szymkowiak , Z. Borowski , W. Kantorowicz , J. M. Espelta , and M. Fernández‐Martínez . 2020. “Investigating the Relationship between Climate, Stand Age, and Temporal Trends in Masting Behavior of European Forest Trees.” Global Change Biology 26(3): gcb.14945. 10.1111/gcb.14945. PMC7079002 31950581

[ecy70076-bib-0045] Petry, W. 2021. “Clone of Digital Representations of Tree Species Range Maps from ‘Atlas of United States Trees’ by Elbert L. Little, Jr. (And other publications).” https://github.com/wpetry/USTreeAtlas.

[ecy70076-bib-0046] Pironon, S. , G. Papuga , J. Villellas , A. L. Angert , M. B. García , and J. D. Thompson . 2017. “Geographic Variation in Genetic and Demographic Performance: New Insights from an Old Biogeographical Paradigm.” Biological Reviews 92: 1877–1909. 27891813 10.1111/brv.12313

[ecy70076-bib-0047] R Core Team . 2023. R: A Language and Environment for Statistical Computing. Vienna: R Foundation for Statistical Computing.

[ecy70076-bib-0048] Roland, C. , J. Schmidt , and J. Johnstone . 2014. “Climate Sensitivity of Reproduction in a Mast Seeding Boreal Conifer across its Distributional Range from Lowland to Treeline Forests.” Oecologia 174: 665–677. 24213628 10.1007/s00442-013-2821-6

[ecy70076-bib-0049] Satake, A. , and O. N. Bjørnstad . 2008. “A Resource Budget Model to Explain Intraspecific Variation in Mast Reproductive Dynamics.” Ecological Research 23: 3–10.

[ecy70076-bib-0050] Sexton, J. P. , P. J. McIntyre , A. L. Angert , and K. J. Rice . 2009. “Evolution and Ecology of Species Range Limits.” Annual Review of Ecology, Evolution, and Systematics 40: 415–436.

[ecy70076-bib-0051] Sharma, S. , R. Andrus , Y. Bergeron , M. Bogdziewicz , D. Bragg , D. Brockway , N. Cleavitt , et al. 2022. “North American Tree Migration Paced by Climate in the West, Lagging in the East.” Proceedings of the National Academy of Sciences of the United States of America 119: e2116691118. 34983867 10.1073/pnas.2116691118 PMC8784119

[ecy70076-bib-0052] Sork, V. L. , J. Bramble , and O. Sexton . 1993. “Ecology of Mast‐Fruiting in Three Species of North American Deciduous Oaks.” Ecology 74: 528–541.

[ecy70076-bib-0053] Touzot, L. , É. Schermer , S. Venner , S. Delzon , C. Rousset , É. Baubet , J.‐M. Gaillard , and M. Gamelon . 2020. “How Does Increasing Mast Seeding Frequency Affect Population Dynamics of Seed Consumers? Wild Boar as a Case Study.” Ecological Applications 30: e02134. 32299142 10.1002/eap.2134

[ecy70076-bib-0054] Van de Pol, M. , and J. Wright . 2009. “A Simple Method for Distinguishing Within‐ Versus Between‐Subject Effects Using Mixed Models.” Animal Behaviour 77: 753–758.

[ecy70076-bib-0055] Vaupel, A. , and D. Matthies . 2012. “Abundance, Reproduction, and Seed Predation of an Alpine Plant Decrease from the Center toward the Range Limit.” Ecology 93: 2253–2262. 23185886 10.1890/11-2026.1

[ecy70076-bib-0056] Waller, D. M. 1979. “Models of Mast Fruiting in Trees.” Journal of Theoretical Biology 80: 223–232. 529801 10.1016/0022-5193(79)90207-8

[ecy70076-bib-0057] Westerband, A. C. , J. L. Funk , and K. E. Barton . 2021. “Intraspecific Trait Variation in Plants: A Renewed Focus on Its Role in Ecological Processes.” Annals of Botany 127: 397–410. 33507251 10.1093/aob/mcab011 PMC7988520

[ecy70076-bib-0058] Willi, Y. , and J. Van Buskirk . 2022. “A Review on Trade‐Offs at the Warm and Cold Ends of Geographical Distributions.” Philosophical Transactions of the Royal Society B: Biological Sciences 377: 20210022. 10.1098/rstb.2021.0022 PMC8859520 35184594

[ecy70076-bib-0059] Wion, A. P. , I. S. Pearse , K. C. Rodman , T. T. Veblen , and M. D. Redmond . 2023. “Masting Is Shaped by Tree‐Level Attributes and Stand Structure, More than Climate, in a Rocky Mountain Conifer Species.” Forest Ecology and Management 531: 120794.

[ecy70076-bib-0060] Wion, A. P. , P. J. Weisberg , I. S. Pearse , and M. D. Redmond . 2020. “Aridity Drives Spatiotemporal Patterns of Masting across the Latitudinal Range of a Dryland Conifer.” Ecography 43: 569–580.

[ecy70076-bib-0061] Zwolak, R. , P. Celebias , and M. Bogdziewicz . 2022. “Global Patterns in the Predator Satiation Effect of Masting: A Meta‐Analysis.” Proceedings of the National Academy of Sciences 119: e2105655119. 10.1073/pnas.2105655119 PMC8931228 35254901

