# Supplementary material for: Intraspecific variation in masting across climate gradients is inconsistent with the environmental stress hypothesis
Source: Ecology. 2025 Apr 3;106(4):e70076. doi: 10.1002/ecy.70076 (PMC11969062; doi:10.1002/ecy.70076)
Supplement: Supplementary file 1 — Appendix S1. [file ECY-106-e70076-s001.pdf]

Appendix S1: Supplementary information

Manuscript title: Intraspecific variation in masting across climate gradients is inconsistent with the environmental stress hypothesis

Author names: Jessie J. Foest, Thomas Caignard, Ian S. Pearse, Michał Bogdziewicz, Andrew Hacket-Pain

Views and opinions expressed are those of the authors only and do not necessarily reflect those of the European Union or the European Research Council. Neither the European Union nor the granting authority can be held responsible for them. Any use of trade, firm, or product names is for descriptive purposes only and does not imply endorsement by the U.S. Government.

Fig. S1

See following pages.

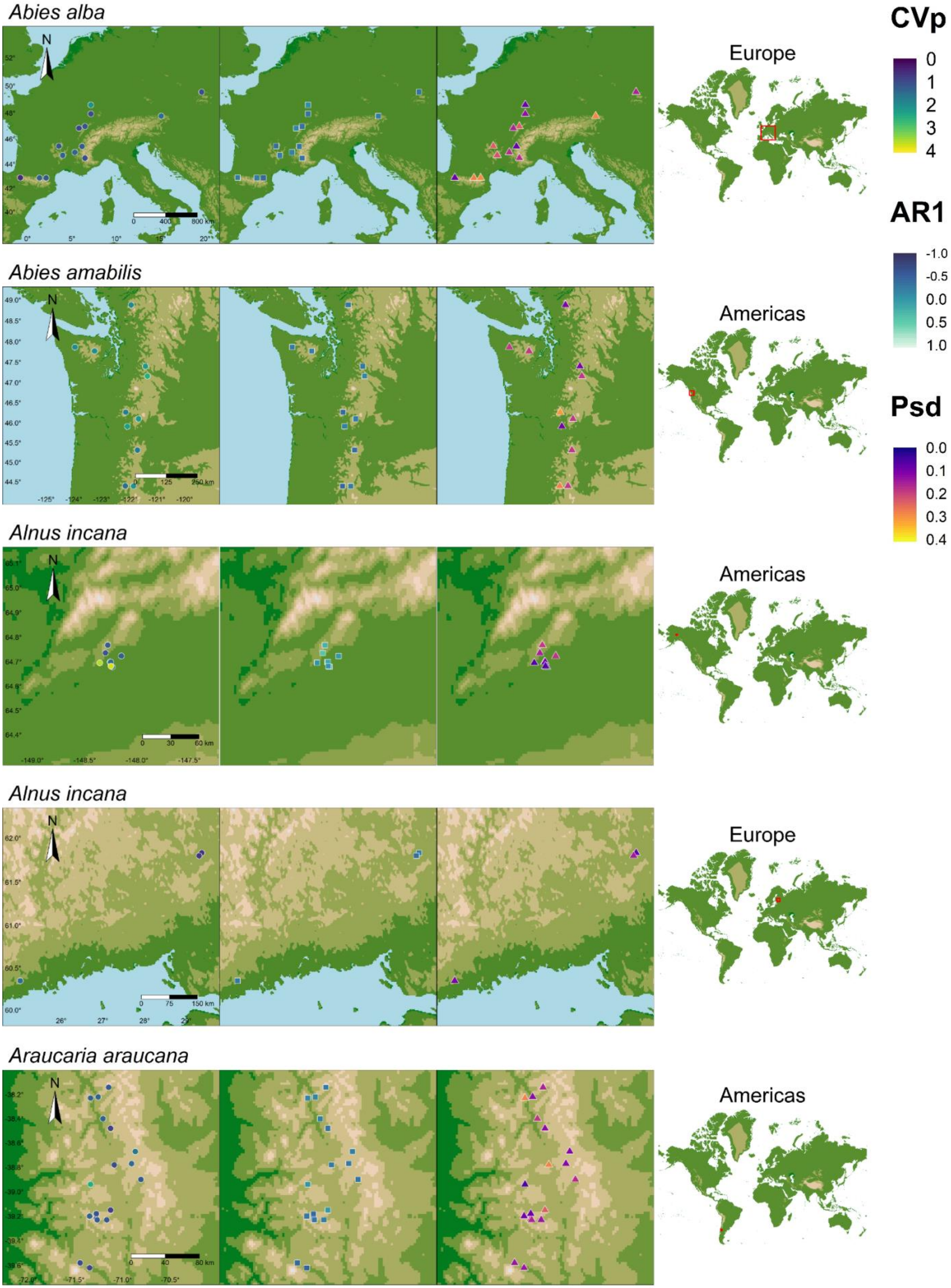
Abies alba
Europe
Abies amabilis
Americas
Alnus incana
Americas
Alnus incana
Europe
Araucaria araucana
Americas
CVp
0
1
2
3
4
AR1
-1.0
-0.5
0.0
0.5
1.0
Psd
0.0
0.1
0.2
0.3
0.4
N
0 400 800 km
0 125 250 km
0 30 60 km
0 75 150 km
0 40 80 km

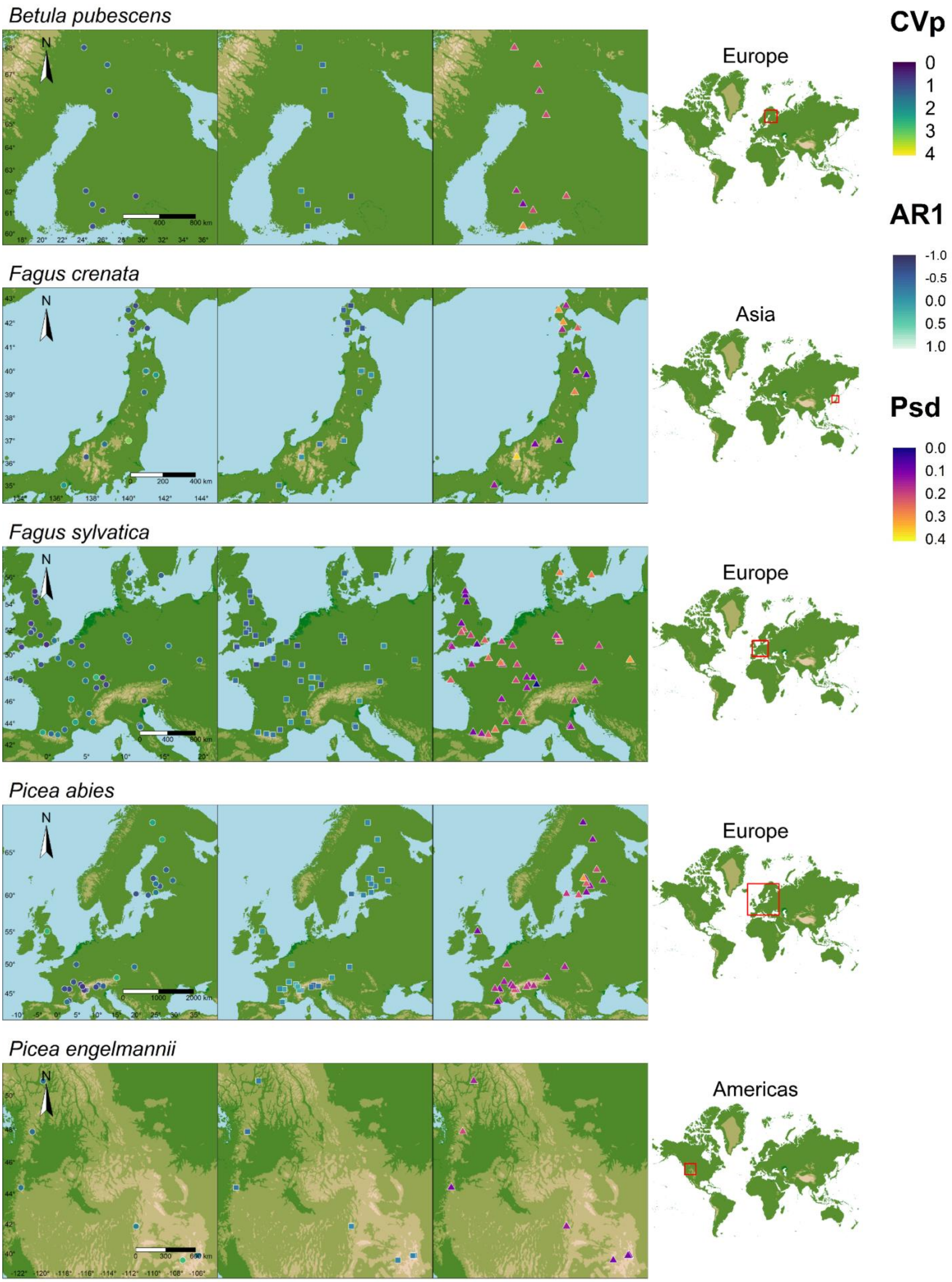

Betula pubescens
Europe
Fagus crenata
Asia
Fagus sylvatica
Europe
Picea abies
Europe
Picea engelmannii
Americas
CVp
0
1
2
3
4
AR1
-1.0
-0.5
0.0
0.5
1.0
Psd
0.0
0.1
0.2
0.3
0.4

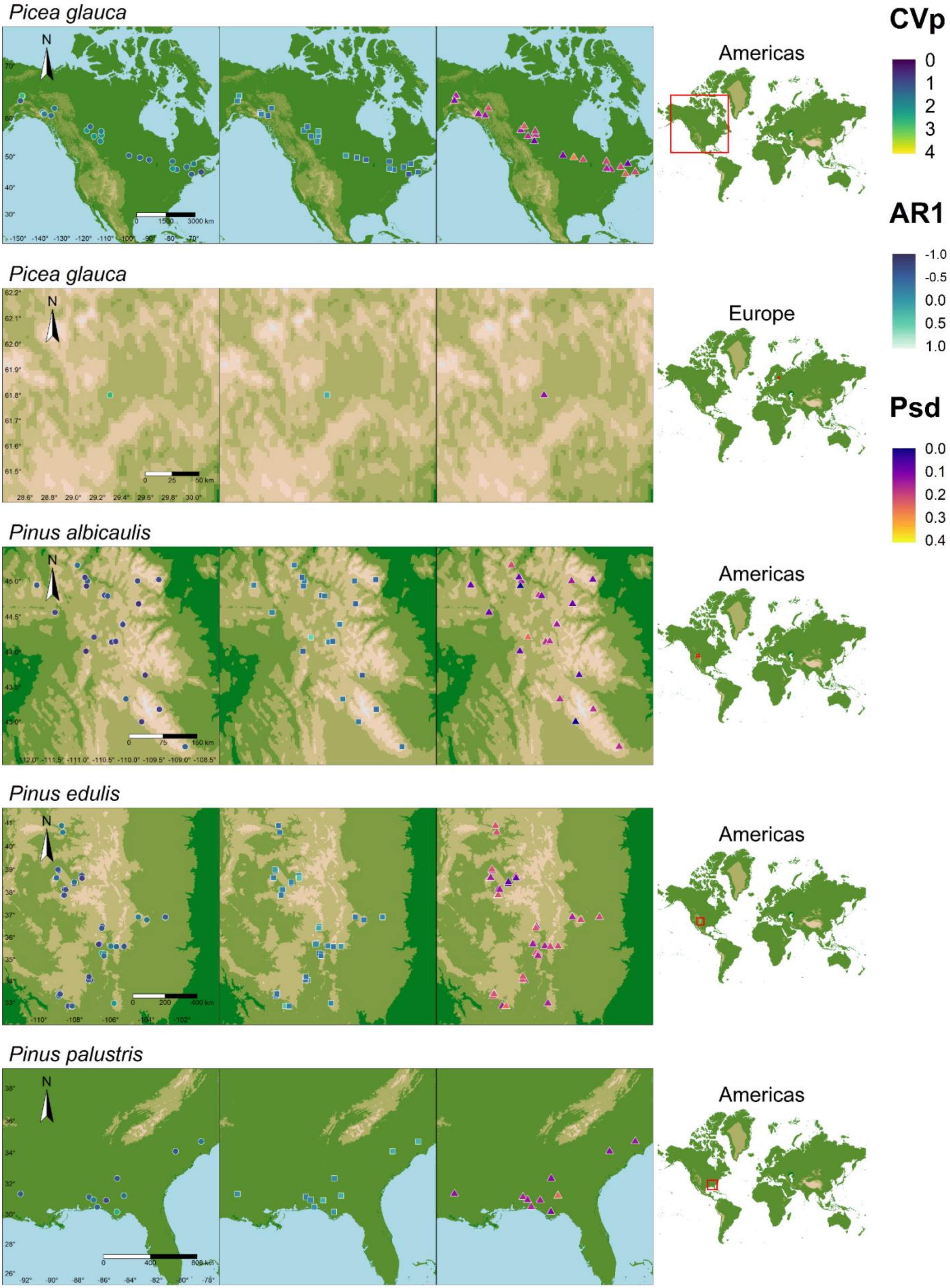

Picea glauca
Americas
Picea glauca
Europe
Pinus albicaulis
Americas
Pinus edulis
Americas
Pinus palustris
Americas
CVp
0
1
2
3
4
AR1
-1.0
-0.5
0.0
0.5
1.0
Psd
0.0
0.1
0.2
0.3
0.4
N

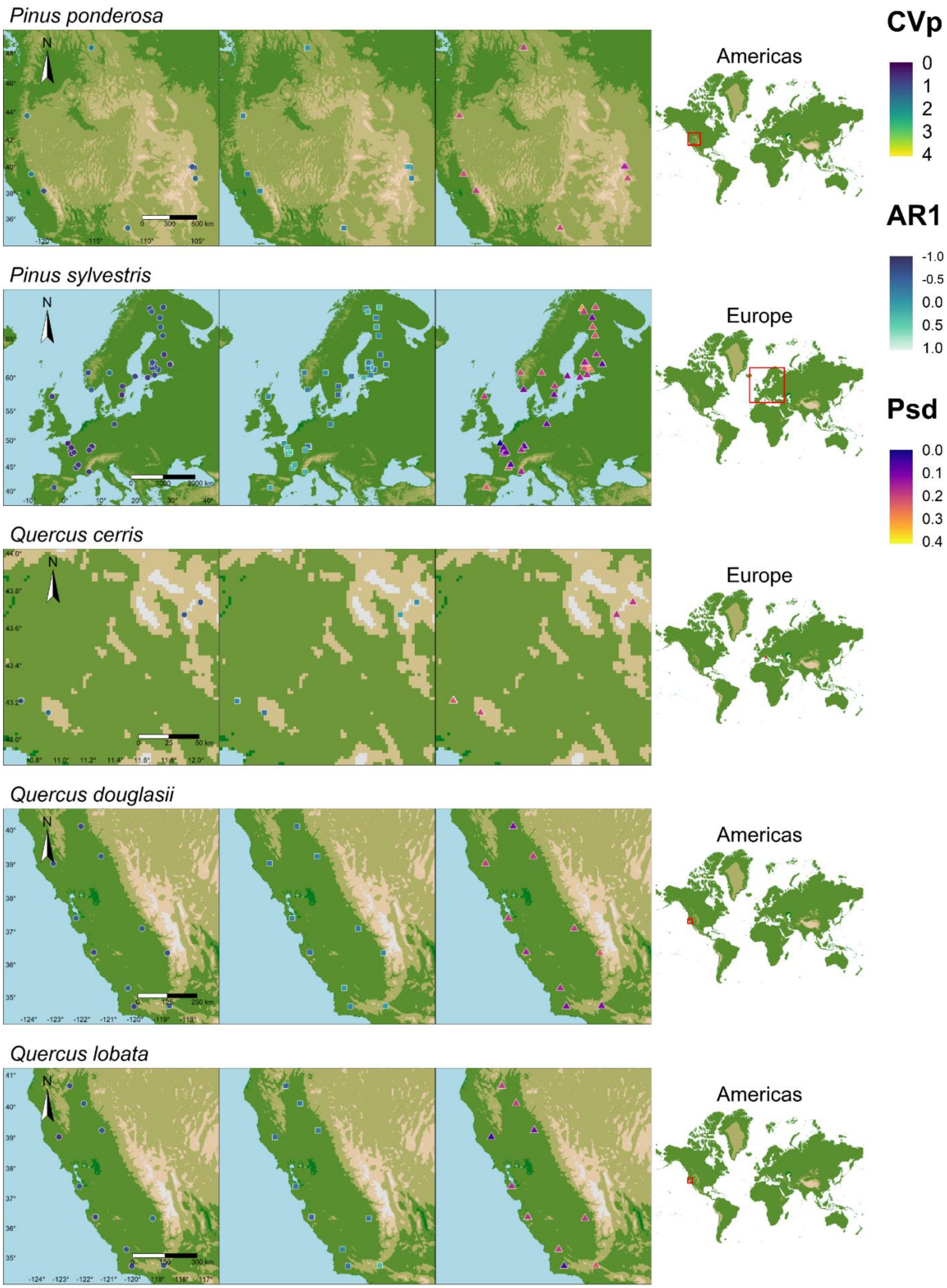
Pinus ponderosa
Americas
Pinus sylvestris
Europe
Quercus cerris
Europe
Quercus douglasii
Americas
Quercus lobata
Americas
CVp
0
1
2
3
4
AR1
-1.0
-0.5
0.0
0.5
1.0
Psd
0.0
0.1
0.2
0.3
0.4
0 300 600 km
0 1000 2000 km
0 25 50 km
0 125 250 km
0 150 300 km

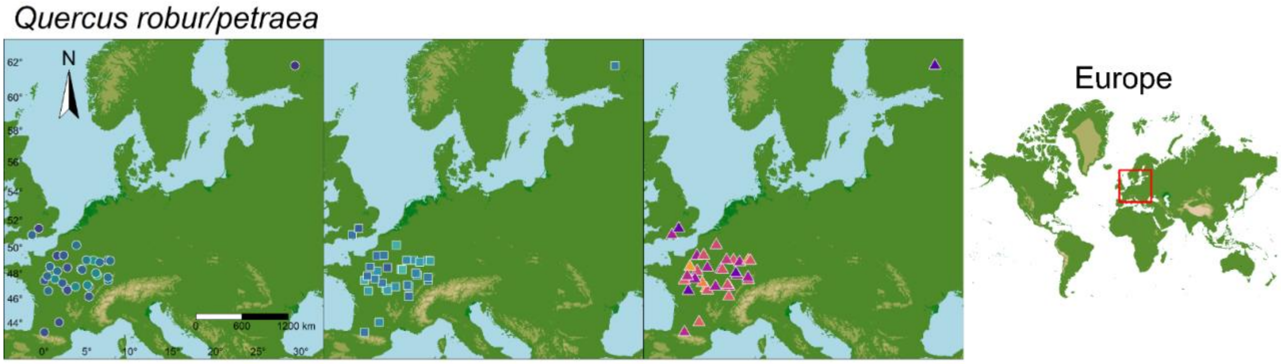

Figure S1: Spatial range and reproductive patterns of all but the six most sampled species in our subset of MASTREE+ data (Hacket-Pain et al. 2022*). When records originate from multiple regions, plots were made for each region. Basemaps were constructed with rnaturalearth (Massicotte & South, 2023) and terrain tiles obtained with elevatr (Hollister et al., 2023; Mapzen 2023). The three metrics used to capture intraspecific variation in masting were CVp: coefficient of variation of population level seed crop size] [left column]; AR(1) =1-year lagged autocorrelation [middle column]; Psd = the proportion of large seed production years [right column].*

Fig. S2

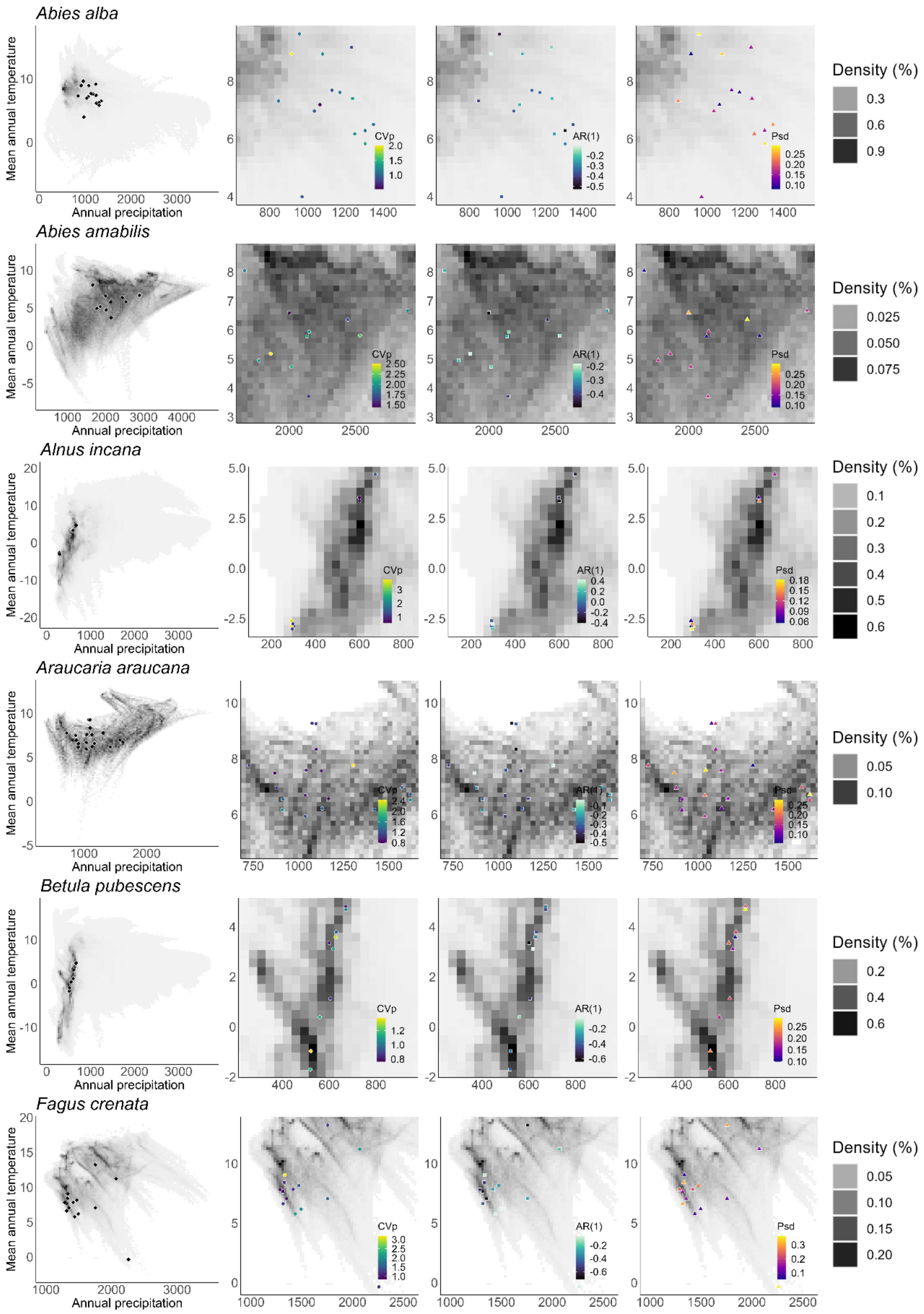

Abies alba
Abies amabilis
Alnus incana
Araucaria araucana
Betula pubescens
Fagus crenata
Mean annual temperature
Annual precipitation
CVp
AR(1)
Psd
Density (%)

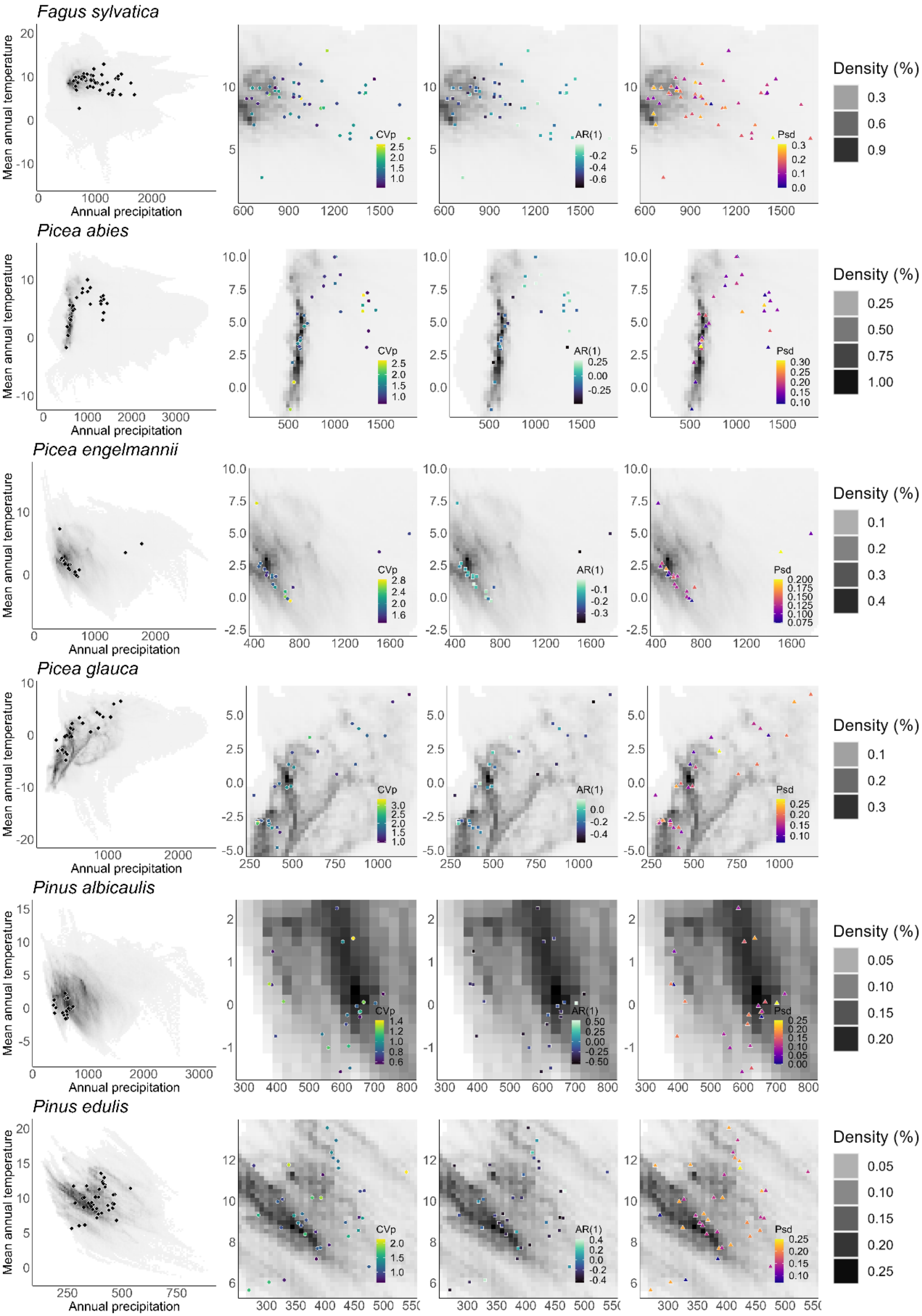
Fagus sylvatica
Mean annual temperature
Annual precipitation
CVp
2.5
2.0
1.5
1.0
AR(1)
-0.2
-0.4
-0.6
Psd
0.3
0.2
0.1
0.0
Density (%)
0.3
0.6
0.9
Picea abies
Mean annual temperature
Annual precipitation
CVp
2.5
2.0
1.5
1.0
AR(1)
0.25
0.00
-0.25
Psd
0.30
0.25
0.20
0.15
0.10
Density (%)
0.25
0.50
0.75
1.00
Picea engelmannii
Mean annual temperature
Annual precipitation
CVp
2.8
2.4
2.0
1.6
AR(1)
-0.1
-0.2
-0.3
Psd
0.200
0.175
0.150
0.125
0.100
0.075
Density (%)
0.1
0.2
0.3
0.4
Picea glauca
Mean annual temperature
Annual precipitation
CVp
3.0
2.5
2.0
1.5
1.0
AR(1)
0.0
-0.2
-0.4
Psd
0.25
0.20
0.15
0.10
Density (%)
0.1
0.2
0.3
Pinus albicaulis
Mean annual temperature
Annual precipitation
CVp
1.4
1.2
1.0
0.8
0.6
AR(1)
0.50
0.25
0.00
-0.25
-0.50
Psd
0.25
0.20
0.15
0.10
0.05
0.00
Density (%)
0.05
0.10
0.15
0.20
Pinus edulis
Mean annual temperature
Annual precipitation
CVp
2.0
1.5
1.0
AR(1)
0.4
0.2
0.0
-0.2
-0.4
Psd
0.25
0.20
0.15
0.10
Density (%)
0.05
0.10
0.15
0.20
0.25

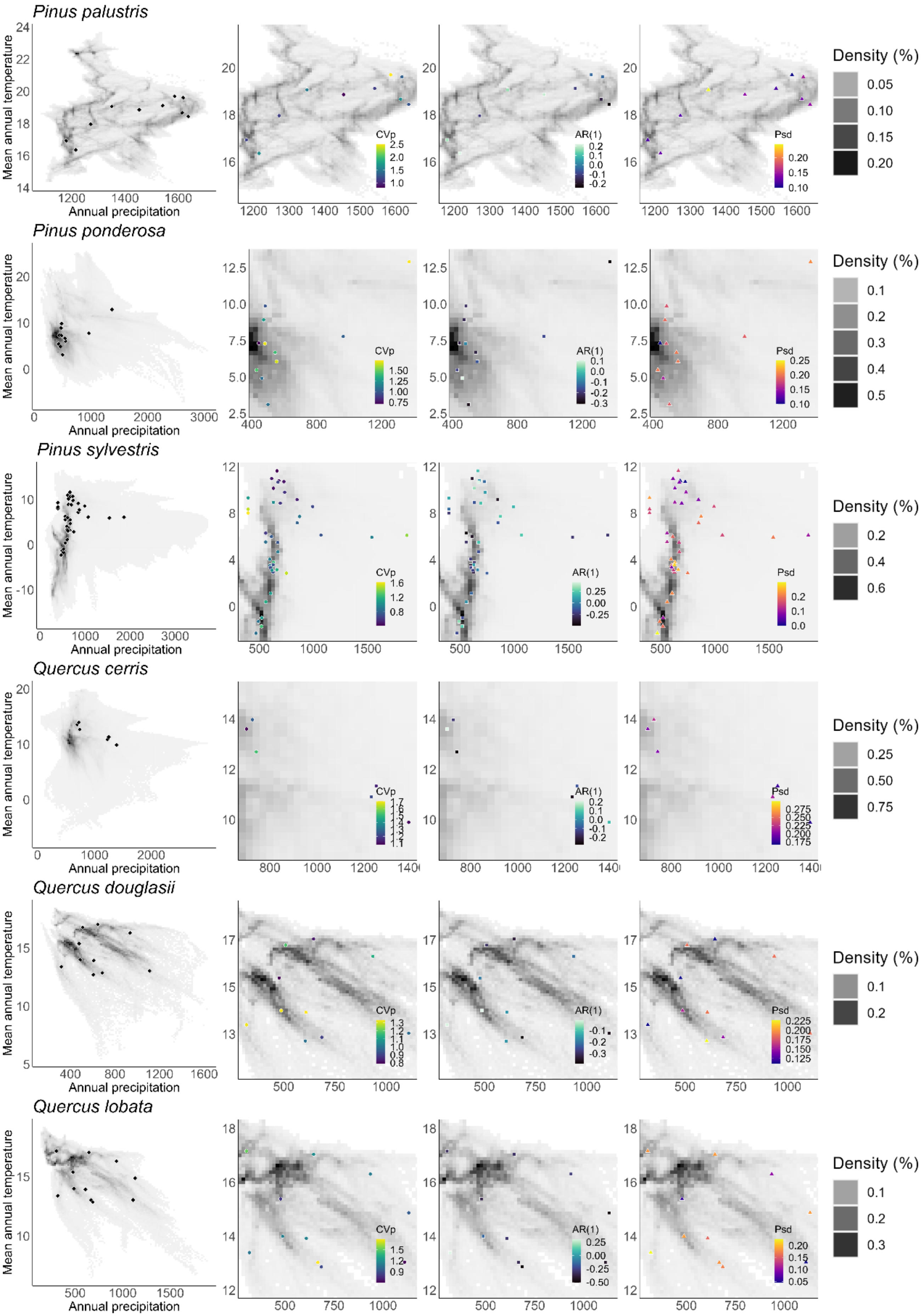

Pinus palustris
Mean annual temperature
Annual precipitation
CVp
AR(1)
Psd
Density (%)
0.05
0.10
0.15
0.20
Pinus ponderosa
Density (%)
0.1
0.2
0.3
0.4
0.5
Pinus sylvestris
Density (%)
0.2
0.4
0.6
Quercus cerris
Density (%)
0.25
0.50
0.75
Quercus douglasii
Density (%)
0.1
0.2
Quercus lobata
Density (%)
0.1
0.2
0.3

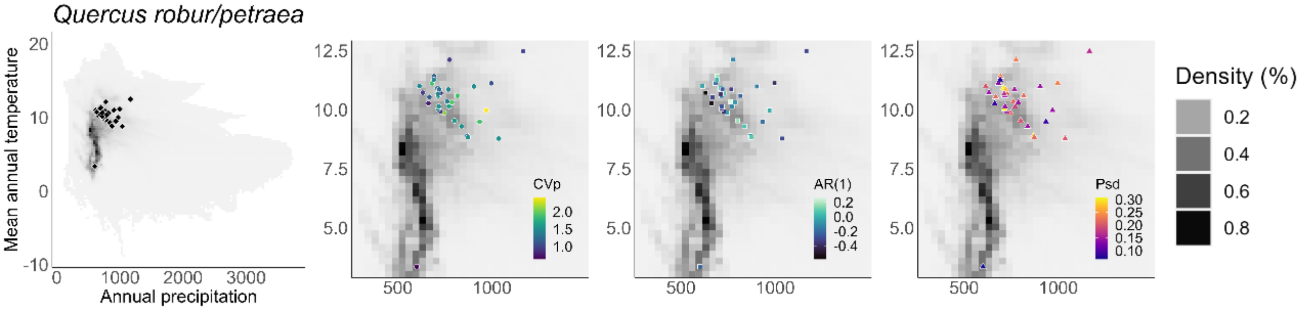

Figure S2: Large climatic gradients were sampled. Points: time series from our subset of MASTREE+ (Hacket-Pain et al. 2022). Plots show the relationships between the reproductive metrics and climate (mean annual temperature in degree Celsius, mean annual precipitation in mm) observed in species' ranges. See Materials and Methods for details about these ranges, but note that shaded areas do not equate to confirmed species presences. Rather, they correspond to grid cells which fall within a geographical distribution of the species – the darker grid cells indicate that the climate is more common in the species range, and may be a better indicator of species presence. CVp: coefficient of variation of population-level seed crop size; AR(1) =1-year lagged autocorrelation; Psd = the proportion of large seed production years.

Fig. S3

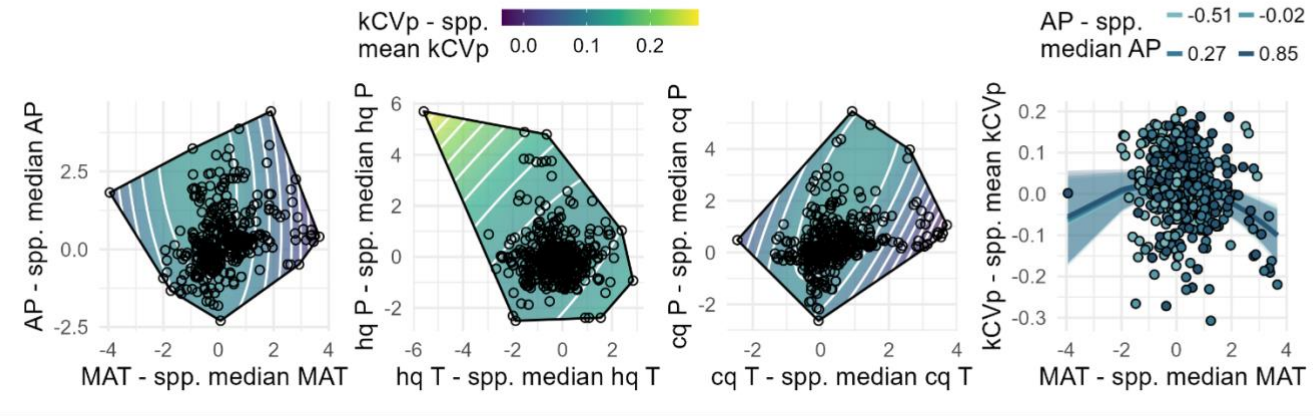

Figure S3: Response surfaces for the subject-centred models of kCVp, a bounded alternative to the coefficient of variation of population level seed crop size (CVp). kCVp is reported to be less sensitive to excess zero values than CVp and was found to increase statistical power (Lobry et al. 2023). The annual model is found in the first column, and the results of the seasonal model are found in columns 2-3. The right-most column shows the marginal effects and partial residuals. AP: annual precipitation; MAT: mean annual temperature; hq: hottest quarter of the year; cq: coldest quarter of the year; P: precipitation; T: temperature.

Table S1

Table S1: Intraspecific variation in reproductive metrics, across tree species.

	Time series		Sites			CVp		AR(1)		Psd	
**Species**	**N**	**Years**	**Latitude**	**Longitude**	**Elevation**	**Mean**	**Range**	**Mean**	**Range**	**Mean**	**Range**
Abies alba	15	1994 – 2018	42.86 – 49.55	-0.8 – 19.61	400 – 1400	1.12 (0.33)	0.52 – 2	-0.32 (0.11)	-0.53 – -0.1	0.18 (0.07)	0.08 – 0.29
Abies amabilis	12	1961 – 1972	44.39 – 48.88	-123.96 – -121.35	423 – 1561	1.94 (0.29)	1.44 – 2.5	-0.3 (0.1)	-0.49 – -0.18	0.18 (0.06)	0.09 – 0.3
Alnus incana	12	1960 – 2008	60.35 – 64.77	-148.36 – 29.37	47 – 404	1.7 (1.02)	0.56 – 3.75	0 (0.26)	-0.4 – 0.42	0.11 (0.05)	0.06 – 0.18
Araucaria araucana	21	1982 – 2020	-39.62 – -38.14	-71.45 – -70.81	1000 – 1698	1.23 (0.37)	0.79 – 2.45	-0.28 (0.12)	-0.51 – -0.03	0.15 (0.06)	0.06 – 0.28
Betula pubescens	14	1955 – 1974	60.33 – 68.02	24.15 – 29.3	47 – 320	1.04 (0.19)	0.77 – 1.38	-0.39 (0.16)	-0.64 – -0.06	0.2 (0.04)	0.1 – 0.29
Fagus crenata	20	1976 – 2017	35.02 – 42.7	136.43 – 141.5	230 – 2815	1.44 (0.55)	0.83 – 3.2	-0.35 (0.2)	-0.71 – -0.06	0.18 (0.09)	0.06 – 0.36
Fagus sylvatica	52	1967 – 2020	42.93 – 56.29	-3.54 – 19.53	26 – 1400	1.35 (0.5)	0.56 – 2.63	-0.37 (0.18)	-0.74 – -0.01	0.17 (0.06)	0 – 0.31
Picea abies	31	1954 – 2018	43.44 – 68.02	-2.63 – 29.3	30 – 2040	1.39 (0.55)	0.66 – 2.63	-0.1 (0.2)	-0.5 – 0.27	0.17 (0.06)	0.09 – 0.31
Picea engelmannii	24	1947 – 2014	39.55 – 50.83	-121.85 – -105.86	1444 – 3474	1.85 (0.41)	1.32 – 2.83	-0.15 (0.09)	-0.39 – -0.02	0.12 (0.03)	0.07 – 0.2
Picea glauca	38	1954 – 2020	44.52 – 64.77	-149.01 – 29.3	9 – 974	1.82 (0.48)	0.93 – 3.34	-0.2 (0.15)	-0.54 – 0.17	0.16 (0.05)	0.07 – 0.27
Pinus albicaulis	22	1989 – 2020	42.64 – 45.22	-111.82 – -108.89	2144 – 3117	0.91 (0.26)	0.56 – 1.41	-0.28 (0.22)	-0.56 – 0.53	0.12 (0.06)	0 – 0.25
Pinus edulis	38	1969 – 2017	32.81 – 40.87	-109.05 – -102.94	1295 – 2388	1.23 (0.37)	0.64 – 2.15	-0.17 (0.21)	-0.45 – 0.42	0.17 (0.05)	0.07 – 0.25
Pinus palustris	10	1958 – 2019	30.15 – 34.71	-92.41 – -78.56	19 – 70	1.41 (0.46)	0.82 – 2.51	0.01 (0.17)	-0.24 – 0.22	0.14 (0.04)	0.1 – 0.25
Pinus ponderosa	12	1933 – 2008	35.27 – 48.37	-121.64 – -105.12	732 – 2797	1.3 (0.33)	0.71 – 1.74	-0.17 (0.11)	-0.31 – 0.1	0.19 (0.04)	0.1 – 0.25
Pinus sylvestris	50	1954 – 2019	40.78 – 68.47	-3.67 – 29.37	24 – 1670	0.89 (0.28)	0.41 – 1.63	-0.09 (0.23)	-0.49 – 0.45	0.16 (0.07)	0 – 0.3
Quercus cerris	11	1992 – 2020	43.14 – 43.74	10.7 – 12.04	299 – 977	1.3 (0.21)	1.08 – 1.71	-0.11 (0.15)	-0.28 – 0.21	0.21 (0.04)	0.17 – 0.3
Quercus douglasii	10	1980 – 2020	34.72 – 40.1	-123.09 – -118.71	120 – 1258	1.08 (0.21)	0.79 – 1.33	-0.21 (0.14)	-0.39 – -0.01	0.16 (0.04)	0.11 – 0.23
Quercus lobata	12	1970 – 2020	34.72 – 40.66	-123.09 – -118.71	120 – 1255	1.13 (0.33)	0.61 – 1.79	-0.3 (0.21)	-0.51 – 0.31	0.15 (0.06)	0.05 – 0.23
Quercus robur/petraea	33	1960 – 2017	43.2 – 61.8	-1.41 – 29.3	43 – 525	1.41 (0.39)	0.63 – 2.41	-0.16 (0.2)	-0.58 – 0.26	0.18 (0.06)	0.07 – 0.31

Information in the table was obtained from the open-access MASTREE+ database (Hacket-Pain et al. 2022). SRTM data (30 sec) was used to estimate missing elevation data in MASTREE+ (57.7% of sites) (Fick and Hijmans 2017).CVp: coefficient of variation of population-level seed crop size; AR(1): 1-year lagged autocorrelation; Psd: the proportion of large seed production years. N: number of time series. Values in brackets are standard deviations. Coordinates are given in decimal degrees, and elevation in meters.

Table S2

Table S2: Likelihood ratio tests for the linear predictors ('Term') in the across-species models, for three masting metrics: CVp, AR(1), and Psd. Bold font added for significant predictors.

Metric	Term	Model	Df	AIC	Deviance	Chisq	Pr(>Chisq)
CVp	AP	Reduced	31	359.101	297.101		
	AP	Full	32	360.628	296.628	0.473	0.492
	MAT	Reduced	31	378.581	316.581		
	**MAT**	**Full**	32	361.f603	297.603	18.978	**> 0.001**
	cq P	Reduced	36	367.022	295.022		
	cq P	Full	37	368.343	294.343	0.68	0.41
	cq T	Reduced	36	379.91	307.91		
	**cq T**	**Full**	37	368.253	294.253	13.657	**> 0.001**
	hq P	Reduced	36	362.405	290.405		
	hq P	Full	37	364.266	290.266	0.14	0.709
	hq T	Reduced	36	362.048	290.048		
	hq T	Full	37	363.89	289.89	0.157	0.692
AR(1)	AP	Reduced	32	-308.706	-372.706		
	AP	Full	33	-308.33	-374.33	1.624	0.203
	MAT	Reduced	32	-297.412	-361.412		
	**MAT**	**Full**	33	-308.005	-374.005	12.594	**> 0.001**
	cq P	Reduced	37	-312.785	-386.785		
	cq P	Full	38	-312.186	-388.186	1.402	0.236
	cq T	Reduced	37	-307.925	-381.925		
	**cq T**	**Full**	38	-315.623	-391.623	9.698	**0.002**
	hq P	Reduced	37	-308.496	-382.496		
	hq P	Full	38	-306.595	-382.595	0.099	0.753
	hq T	Reduced	37	-308.896	-382.896		
	hq T	Full	38	-307.216	-383.216	0.321	0.571
Psd	AP	Reduced	31	-1277.077	-1339.077		
	AP	Full	32	-1276.322	-1340.322	1.245	0.265
	MAT	Reduced	31	-1275.592	-1337.592		
	MAT	Full	32	-1275.588	-1339.588	1.995	0.158
	cq P	Reduced	36	-1267.286	-1339.286		
	cq P	Full	37	-1266.487	-1340.487	1.201	0.273
	cq T	Reduced	36	-1267.286	-1339.286		
	cq T	Full	37	-1266.487	-1340.487	1.201	0.273
	hq P	Reduced	36	-1274.675	-1346.675		
	hq P	Full	37	-1273.178	-1347.178	0.503	0.478
	hq T	Reduced	36	-1276.432	-1348.432		
	hq T	Full	37	-1274.659	-1348.659	0.227	0.634

CVp: coefficient of variation of population level seed crop size; AR(1): 1-year lagged autocorrelation; Psd: the proportion of large seed production years.

Table S3

Table S3: Full models for subject-centred annual and seasonal models for three masting metrics, CVp, AR(1), Psd, as well as kCVp.

	CVp			AR(1)			Psd			kCVp		
**Term**	**Estimate**	**z**	**P**	**Estimate**	**z**	**P**	**Estimate**	**z**	**P**	**Estimate**	**z**	**P**
Annual												
Intercept	**6.01×10^{-1}<br>(2.58×10^{-1})**	**2.33**	**0.02**	5.87×10^{-2}<br>(7.81×10^{-2})	0.75	0.45	5.33×10^{-2}<br>(3.75×10^{-2})	1.42	0.16	**1.13×10^{-1}<br>(4.85×10^{-2})**	**2.33**	**0.02**
AP	9.73×10^{-3}<br>(3.07×10^{-2})	0.32	0.75	1.49×10^{-3}<br>(1.29×10^{-2})	0.12	0.91	**9.67×10^{-3}<br>(4.51×10^{-3})**	**2.14**	**0.03**	4.88×10^{-3}<br>(5.84×10^{-3})	0.84	0.4
AP^2	-1.40×10^{-3}<br>(1.38×10^{-2})	-0.10	0.92	-2.86×10^{-3}<br>(5.32×10^{-3})	-0.54	0.59	-3.75×10^{-3}<br>(2.03×10^{-3})	-1.84	0.07	-1.18×10^{-3}<br>(2.66×10^{-3})	-0.44	0.66
MAT	**-6.21×10^{-2}<br>(2.69×10^{-2})**	**-2.31**	**0.02**	**-3.45×10^{-2}<br>(1.10×10^{-2})**	**-3.13**	**<0.01**	-1.31×10^{-3}<br>(4.08×10^{-3})	-0.32	0.75	-6.64×10^{-3}<br>(5.64×10^{-3})	-1.18	0.24
MAT × AP	1.43×10^{-2}<br>(1.95×10^{-2})	0.73	0.47	-1.51×10^{-2}<br>(9.75×10^{-3})	-1.55	0.12	2.37×10^{-3}<br>(3.55×10^{-3})	0.67	0.5	-1.60×10^{-3}<br>(4.45×10^{-3})	-0.36	0.72
MAT^2	-8.66×10^{-3}<br>(8.77×10^{-3})	-0.99	0.32	9.95×10^{-3}<br>(5.95×10^{-3})	1.67	0.09	**-4.23×10^{-3}<br>(2.06×10^{-3})**	**-2.05**	**0.04**	**-6.99×10^{-3}<br>(2.53×10^{-3})**	**-2.77**	**<0.01**
Latitude				**-2.50×10^{-2}<br>(8.93×10^{-3})**	**-2.80**	**<0.01**						
Length	2.63×10^{-2}<br>(2.06×10^{-2})	1.27	0.2	**1.50×10^{-2}<br>(6.01×10^{-3})**	**2.49**	**0.01**	**-4.99×10^{-3}<br>(2.33×10^{-3})**	**-2.14**	**0.03**	4.54×10^{-3}<br>(3.52×10^{-3})	1.29	0.2
Method: Count	**-6.39×10^{-1}<br>(2.54×10^{-1})**	**-2.51**	**0.01**	-7.83×10^{-2}<br>(7.50×10^{-2})	-1.05	0.3	-4.79×10^{-2}<br>(3.69×10^{-2})	-1.30	0.19	**-1.13×10^{-1}<br>(4.79×10^{-2})**	**-2.35**	**0.02**
Method: Seed trap	-2.38×10^{-1}<br>(2.39×10^{-1})	-1.00	0.32	-2.21×10^{-2}<br>(6.95×10^{-2})	-0.32	0.75	-3.78×10^{-2}<br>(3.59×10^{-2})	-1.05	0.29	-4.88×10^{-2}<br>(4.47×10^{-2})	-1.09	0.27
Variable: Fruit	**-4.35×10^{-1}<br>(9.82×10^{-2})**	**-4.43**	**<0.001**	9.84×10^{-3}<br>(4.03×10^{-2})	0.24	0.81	-2.31×10^{-2}<br>(1.23×10^{-2})	-1.87	0.06	**-8.49×10^{-2}<br>(2.21×10^{-2})**	**-3.84**	**<0.001**
Variable: Seed	**-2.43×10^{-1}<br>(8.14×10^{-2})**	**-2.99**	**<0.01**	-2.61×10^{-2}<br>(2.87×10^{-2})	-0.91	0.36	-8.84×10^{-3}<br>(8.76×10^{-3})	-1.01	0.31	**-3.79×10^{-2}<br>(1.63×10^{-2})**	**-2.32**	**0.02**
Seasonal												
Intercept	**6.58×10^{-1}<br>(2.58×10^{-1})**	**2.55**	**0.01**	1.24×10^{-1}<br>(7.82×10^{-2})	1.58	0.11	4.91×10^{-2}<br>(3.82×10^{-2})	1.29	0.2	**1.13×10^{-1}<br>(4.87×10^{-2})**	**2.32**	**0.02**
hq P	-4.08×10^{-3}<br>(2.77×10^{-2})	-0.15	0.88	2.29×10^{-3}<br>(1.09×10^{-2})	0.21	0.83	-1.74×10^{-3}<br>(3.81×10^{-3})	-0.46	0.65	-3.95×10^{-4}<br>(5.35×10^{-3})	-0.07	0.94
hq P^2	6.14×10^{-3}<br>(1.22×10^{-2})	0.50	0.61	**-1.14×10^{-2}<br>(3.98×10^{-3})**	**-2.87**	**<0.01**	1.35×10^{-3}<br>(1.60×10^{-3})	0.84	0.4	1.33×10^{-3}<br>(2.32×10^{-3})	0.57	0.57
hq T	5.15×10^{-3}<br>(2.44×10^{-2})	0.21	0.83	2.32×10^{-4}<br>(8.66×10^{-3})	0.03	0.98	-1.07×10^{-3}<br>(3.21×10^{-3})	-0.33	0.74	1.43×10^{-3}<br>(4.64×10^{-3})	0.31	0.76
hq T × hq P	-4.49×10^{-3}<br>(1.83×10^{-2})	-0.25	0.81	**-2.18×10^{-2}<br>(7.17×10^{-3})**	**-3.05**	**<0.01**	-2.91×10^{-3}<br>(2.70×10^{-3})	-1.08	0.28	-2.47×10^{-3}<br>(3.45×10^{-3})	-0.72	0.47
hq T^2	-5.03×10^{-3}<br>(1.33×10^{-2})	-0.38	0.7	-1.79×10^{-3}<br>(5.07×10^{-3})	-0.35	0.72	1.07×10^{-3}<br>(1.91×10^{-3})	0.56	0.58	1.66×10^{-3}<br>(2.57×10^{-3})	0.65	0.52
cq P	1.96×10^{-2}<br>(3.15×10^{-2})	0.62	0.54	1.78×10^{-2}<br>(1.25×10^{-2})	1.43	0.15	6.74×10^{-3}<br>(4.87×10^{-3})	1.39	0.17	5.56×10^{-3}<br>(5.76×10^{-3})	0.97	0.33
cq P^2	-1.36×10^{-2}<br>(1.01×10^{-2})	-1.35	0.18	**-8.32×10^{-3}<br>(3.79×10^{-3})**	**-2.20**	**0.03**	-2.23×10^{-3}<br>(1.53×10^{-3})	-1.46	0.14	-2.40×10^{-3}<br>(1.92×10^{-3})	-1.25	0.21
cq T	**-1.02×10^{-1}<br>(4.45×10^{-2})**	**-2.30**	**0.02**	**-5.96×10^{-2}<br>(1.92×10^{-2})**	**-3.10**	**<0.01**	8.26×10^{-3}<br>(5.98×10^{-3})	1.38	0.17	-1.82×10^{-3}<br>(9.62×10^{-3})	-0.19	0.85
cq T × cq P	**4.46×10^{-2}<br>(2.11×10^{-2})**	**2.11**	**0.03**	8.31×10^{-3}<br>(1.40×10^{-2})	0.59	0.55	3.42×10^{-3}<br>(4.53×10^{-3})	0.75	0.45	8.76×10^{-3}<br>(6.50×10^{-3})	1.35	0.18
cq T^2	-4.68×10^{-3}<br>(1.45×10^{-2})	-0.32	0.75	7.67×10^{-3}<br>(8.76×10^{-3})	0.88	0.38	**-8.13×10^{-3}<br>(2.53×10^{-3})**	**-3.21**	**<0.01**	**-1.29×10^{-2}<br>(3.82×10^{-3})**	**-3.38**	**<0.001**
Latitude				**-2.68×10^{-2}<br>(9.19×10^{-3})**	**-2.92**	**<0.01**						
Length	2.67×10^{-2}<br>(2.07×10^{-2})	1.29	0.2	**1.53×10^{-2}<br>(6.54×10^{-3})**	**2.35**	**0.02**	-4.38×10^{-3}<br>(2.45×10^{-3})	-1.79	0.07	5.61×10^{-3}<br>(3.36×10^{-3})	1.67	0.09
Method: Count	**-6.77×10^{-1}<br>(2.54×10^{-1})**	**-2.66**	**<0.01**	-1.20×10^{-1}<br>(7.55×10^{-2})	-1.59	0.11	-4.71×10^{-2}<br>(3.75×10^{-2})	-1.26	0.21	**-1.15×10^{-1}<br>(4.84×10^{-2})**	**-2.38**	**0.02**
Method: Seed trap	-2.73×10^{-1}<br>(2.36×10^{-1})	-1.16	0.25	-6.55×10^{-2}<br>(6.90×10^{-2})	-0.95	0.34	-3.57×10^{-2}<br>(3.65×10^{-2})	-0.98	0.33	-5.14×10^{-2}<br>(4.47×10^{-2})	-1.15	0.25
Variable: Fruit	**-4.47×10^{-1}<br>(1.01×10^{-1})**	**-4.43**	**<0.001**	-7.47×10^{-3}<br>(4.01×10^{-2})	-0.19	0.85	-2.39×10^{-2}<br>(1.27×10^{-2})	-1.88	0.06	**-7.92×10^{-2}<br>(2.25×10^{-2})**	**-3.53**	**<0.001**
Variable: Seed	**-2.17×10^{-1}<br>(8.81×10^{-2})**	**-2.46**	**0.01**	-2.68×10^{-2}<br>(3.01×10^{-2})	-0.89	0.37	-1.15×10^{-2}<br>(9.26×10^{-3})	-1.25	0.21	**-3.69×10^{-2}<br>(1.70×10^{-2})**	**-2.18**	**0.03**

Columns = CVp: coefficient of variation of population level seed crop size; AR(1): 1-year lagged autocorrelation; Psd: the proportion of large seed production years; kCVp: a bounded alternative to CVp; P: P-value. Rows = AP: annual precipitation; MAT: mean annual temperature; hq: hottest quarter; cq: coldest quarter; P: precipitation; T: temperature. Bolded values indicate significant variables. Shading indicates polynomial predictors. The independent and dependent variables were centred (climate variables: with species' range median value; other variables: species' mean), and subsequently scaled using the root mean square. P-values were calculated with Wald tests via the summary() function. For linear climate predictors, these p-values reflect conditional hypotheses and should not be interpreted as evidence of independent contributions due to the inclusion of quadratic terms as I(x^2).

Table S4

Table S4: Full model results for species-specific models, relating climate gradients to intraspecific variation in masting metrics CVp, AR(1) and Psd.

		F. sylvatica			*P. abies*			*P. edulis*			*P. glauca*			*P. sylvestris*			*Q. robur/petraea*		
**Metric**	**Term**	**Estimate**	**z**	***P***	**Estimate**	**z**	***P***	**Estimate**	**z**	***P***	**Estimate**	**z**	***P***	**Estimate**	**z**	***P***	**Estimate**	**z**	***P***
**CVp**	AP	1.36×10^{-4}<br>(4.55×10^{-4})	0.30	0.77	2.93×10^{-3}<br>(1.37×10^{-3})	2.15	**0.03**	-1.67×10^{-3}<br>(1.19×10^{-3})	-1.40	0.16	-4.23×10^{-4}<br>(9.02×10^{-4})	-0.47	0.64	1.86×10^{-3}<br>(8.96×10^{-4})	2.08	**0.04**	2.09×10^{-3}<br>(1.85×10^{-3})	1.13	0.26
	AP^2	-5.22×10^{-7}<br>(5.99×10^{-7})	-0.87	0.38	-3.70×10^{-6}<br>(1.78×10^{-6})	-2.08	**0.04**	2.26×10^{-5}<br>(1.19×10^{-5})	1.90	0.06	-2.40×10^{-6}<br>(3.38×10^{-6})	-0.71	0.48	-2.44×10^{-7}<br>(4.18×10^{-7})	-0.58	0.56	-3.34×10^{-6}<br>(3.61×10^{-6})	-0.93	0.35
	MAT	-4.04×10^{-2}<br>(4.71×10^{-2})	-0.86	0.39	-1.29×10^{-1}<br>(5.42×10^{-2})	-2.38	**0.02**	1.31×10^{-2}<br>(3.02×10^{-2})	0.44	0.66	1.70×10^{-1}<br>(8.64×10^{-2})	1.96	**0.05**	-5.07×10^{-2}<br>(2.58×10^{-2})	-1.96	**0.05**	3.51×10^{-2}<br>(4.96×10^{-2})	0.71	0.48
	MAT^2	2.17×10^{-2}<br>(9.61×10^{-3})	2.26	**0.02**	1.04×10^{-2}<br>(9.43×10^{-3})	1.11	0.27	-5.67×10^{-3}<br>(1.39×10^{-2})	-0.41	0.68	-2.14×10^{-2}<br>(2.12×10^{-2})	-1.01	0.31	2.08×10^{-3}<br>(3.07×10^{-3})	0.68	0.50	-2.83×10^{-2}<br>(2.84×10^{-2})	-1.00	0.32
	MAT x AP	9.07×10^{-7}<br>(9.73×10^{-5})	0.01	0.99	2.84×10^{-4}<br>(1.57×10^{-4})	1.81	0.07	6.17×10^{-4}<br>(5.33×10^{-4})	1.16	0.25	2.04×10^{-4}<br>(4.73×10^{-4})	0.43	0.67	-1.95×10^{-4}<br>(1.20×10^{-4})	-1.62	0.10	-4.62×10^{-5}<br>(5.66×10^{-4})	-0.08	0.93
	Length	2.73×10^{-2}<br>(1.08×10^{-2})	2.52	**0.01**	-9.24×10^{-3}<br>(7.84×10^{-3})	-1.18	0.24	3.22×10^{-2}<br>(2.18×10^{-2})	1.48	0.14	3.52×10^{-2}<br>(5.48×10^{-3})	6.42	**<0.001**	8.63×10^{-4}<br>(4.72×10^{-3})	0.18	0.85	2.02×10^{-2}<br>(1.81×10^{-2})	1.12	0.26
	Method: Count	-1.39×10^{0}<br>(2.72×10^{-1})	-5.11	**<0.001**	-1.28×10^{0}<br>(3.72×10^{-1})	-3.44	**<0.001**							-8.00×10^{-1}<br>(2.82×10^{-1})	-2.84	**<0.01**	-4.50×10^{-1}<br>(2.94×10^{-1})	-1.53	0.13
	Variable : Fruit				-1.61×10^{0}<br>(3.28×10^{-1})	-4.89	**<0.001**							-8.75×10^{-1}<br>(2.45×10^{-1})	-3.57	**<0.001**			
	Variable : Seed	5.71×10^{-1}<br>(1.49×10^{-1})	3.84	**<0.001**	-6.12×10^{-1}<br>(3.43×10^{-1})	-1.78	0.07				2.68×10^{-1}<br>(1.20×10^{-1})	2.24	**0.03**	-7.27×10^{-1}<br>(1.58×10^{-1})	-4.60	**<0.001**	6.08×10^{-2}<br>(3.62×10^{-1})	0.17	0.87
**AR(1)**	AP	1.62×10^{-4}<br>(1.68×10^{-4})	0.97	0.33	1.57×10^{-3}<br>(2.89×10^{-4})	5.45	**<0.001**	5.64×10^{-4}<br>(7.71×10^{-4})	0.73	0.46	7.88×10^{-5}<br>(3.44×10^{-4})	0.23	0.82	1.56×10^{-3}<br>(1.01×10^{-3})	1.55	0.12	2.40×10^{-3}<br>(1.01×10^{-3})	2.37	**0.02**
	AP^2	1.12×10^{-7}<br>(2.21×10^{-7})	0.51	0.61	-2.15×10^{-6}<br>(3.76×10^{-7})	-5.72	**<0.001**	-3.69×10^{-6}<br>(7.70×10^{-6})	-0.48	0.63	1.48×10^{-7}<br>(1.29×10^{-6})	0.11	0.91	-4.85×10^{-7}<br>(4.70×10^{-7})	-1.03	0.30	-4.07×10^{-6}<br>(1.98×10^{-6})	-2.06	**0.04**
	MAT	-3.57×10^{-2}<br>(1.74×10^{-2})	-2.05	**0.04**	-8.39×10^{-2}<br>(1.15×10^{-2})	-7.32	**<0.001**	-2.62×10^{-3}<br>(1.95×10^{-2})	-0.13	0.89	3.47×10^{-3}<br>(3.30×10^{-2})	0.11	0.92	-9.12×10^{-2}<br>(2.91×10^{-2})	-3.14	**<0.01**	-8.42×10^{-3}<br>(2.72×10^{-2})	-0.31	0.76
	MAT^2	4.26×10^{-3}<br>(3.55×10^{-3})	1.20	0.23	-1.20×10^{-2}<br>(1.99×10^{-3})	-6.01	**<0.001**	3.80×10^{-3}<br>(9.01×10^{-3})	0.42	0.67	9.68×10^{-3}<br>(8.08×10^{-3})	1.20	0.23	7.09×10^{-3}<br>(3.45×10^{-3})	2.05	**0.04**	1.77×10^{-2}<br>(1.56×10^{-2})	1.14	0.26
	MAT x AP	6.24×10^{-5}<br>(3.59×10^{-5})	1.74	0.08	1.69×10^{-4}<br>(3.31×10^{-5})	5.10	**<0.001**	1.16×10^{-4}<br>(3.45×10^{-4})	0.34	0.74	-1.82×10^{-4}<br>(1.80×10^{-4})	-1.01	0.31	-1.49×10^{-4}<br>(1.35×10^{-4})	-1.11	0.27	-2.43×10^{-4}<br>(3.11×10^{-4})	-0.78	0.43
	Length	-4.35×10^{-3}<br>(4.00×10^{-3})	-1.09	0.28	-4.61×10^{-3}<br>(1.66×10^{-3})	-2.78	**<0.01**	-3.95×10^{-3}<br>(1.41×10^{-2})	-0.28	0.78	5.88×10^{-3}<br>(2.09×10^{-3})	2.81	**<0.01**	-6.13×10^{-4}<br>(5.31×10^{-3})	-0.12	0.91	9.45×10^{-4}<br>(9.91×10^{-3})	0.10	0.92
	Method: Count	-1.43×10^{-2}<br>(1.00×10^{-1})	-0.14	0.89	-3.71×10^{-1}<br>(7.86×10^{-2})	-4.71	**<0.001**							-2.13×10^{-1}<br>(3.17×10^{-1})	-0.67	0.50	-2.15×10^{-1}<br>(1.61×10^{-1})	-1.33	0.18

	Variable : Fruit				2.53×10^{-1} (6.93×10^{-2})	3.65	**<0.001**							2.18×10^{-2} (2.75×10^{-1})	0.08	0.94			
	Variable : Seed	2.33×10^{-1} (5.49×10^{-2})	4.24	**<0.001**	-7.63×10^{-2} (7.25×10^{-2})	-1.05	0.29				8.66×10^{-2} (4.56×10^{-2})	1.90	0.06	-3.10×10^{-1} (1.78×10^{-1})	-1.75	0.08	-1.37×10^{-1} (1.99×10^{-1})	-0.69	0.49
**Psd**	AP	-4.13×10^{-5} (7.75×10^{-5})	-0.53	0.59	3.77×10^{-4} (2.17×10^{-4})	1.74	0.08	-1.41×10^{-5} (1.61×10^{-4})	-0.09	0.93	-8.91×10^{-5} (1.40×10^{-4})	-0.64	0.53	4.50×10^{-4} (2.77×10^{-4})	1.62	0.10	5.05×10^{-5} (3.26×10^{-4})	0.15	0.88
	AP^2	-2.53 × 10^-8.00^ (1.02 × 10^{-7})	-0.25	0.80	-5.40×10^{-7} (2.83×10^{-7})	-1.91	0.06	-4.02×10^{-7} (1.61×10^{-6})	-0.25	0.80	-4.70×10^{-7} (5.26×10^{-7})	-0.89	0.37	-2.56×10^{-7} (1.29×10^{-7})	-1.98	**0.05**	-7.84×10^{-7} (6.38×10^{-7})	-1.23	0.22
	MAT	-4.44×10^{-3} (8.03×10^{-3})	-0.55	0.58	-1.19×10^{-2} (8.62×10^{-3})	-1.38	0.17	5.36×10^{-3} (4.06×10^{-3})	1.32	0.19	7.87×10^{-3} (1.34×10^{-2})	0.59	0.56	-1.58×10^{-2} (7.98×10^{-3})	-1.98	**0.05**	1.83×10^{-2} (8.77×10^{-3})	2.09	**0.04**
	MAT^2	-4.71×10^{-4} (1.64×10^{-3})	-0.29	0.77	-3.89×10^{-3} (1.50×10^{-3})	-2.60	**<0.01**	3.84×10^{-4} (1.88×10^{-3})	0.20	0.84	-3.66×10^{-3} (3.29×10^{-3})	-1.11	0.27	5.22×10^{-4} (9.49×10^{-4})	0.55	0.58	-4.77×10^{-3} (5.02×10^{-3})	-0.95	0.34
	MAT x AP	-1.38×10^{-5} (1.66×10^{-5})	-0.84	0.40	4.33×10^{-5} (2.49×10^{-5})	1.74	0.08	1.00×10^{-5} (7.18×10^{-5})	0.14	0.89	8.52×10^{-5} (7.35×10^{-5})	1.16	0.25	-2.15×10^{-5} (3.71×10^{-5})	-0.58	0.56	7.67×10^{-5} (1.00×10^{-4})	0.77	0.44
	Length	-2.87×10^{-3} (1.84×10^{-3})	-1.56	0.12	-9.78×10^{-4} (1.25×10^{-3})	-0.79	0.43	-2.00×10^{-4} (2.94×10^{-3})	-0.07	0.95	-9.58×10^{-4} (8.53×10^{-4})	-1.12	0.26	-1.97×10^{-3} (1.46×10^{-3})	-1.35	0.18	-3.98×10^{-3} (3.19×10^{-3})	-1.25	0.21
	Method: Count	1.59×10^{-3} (4.62×10^{-2})	0.03	0.97	1.04×10^{-1} (5.92×10^{-2})	1.76	0.08							-1.08×10^{-1} (8.72×10^{-2})	-1.24	0.21	-1.02×10^{-1} (5.20×10^{-2})	-1.97	**0.05**
	Variable : Fruit				8.79×10^{-2} (5.22×10^{-2})	1.68	0.09							-1.61×10^{-1} (7.57×10^{-2})	-2.13	**0.03**			
	Variable : Seed	4.37×10^{-2} (2.53×10^{-2})	1.73	0.08	1.08×10^{-1} (5.46×10^{-2})	1.98	**0.05**				-1.80×10^{-2} (1.86×10^{-2})	-0.96	0.33	-1.24×10^{-1} (4.88×10^{-2})	-2.53	**0.01**	8.57×10^{-2} (6.40×10^{-2})	1.34	0.18

Columns = F. sylvatica: Fagus sylvatica; P. abies: Picea abies; P. edulis: Pinus edulis; P. glauca: Picea glauca; P. sylvestris: Pinus sylvestris: Q. robur/petraea: Q. robur/petraea. P: P-value.
Rows= CVp: coefficient of variation of population level seed crop size; AR(1): 1-year lagged autocorrelation; Psd: the proportion of large seed production years; AP: annual precipitation; MAT: mean annual temperature.
Bolded values indicate significant values. Shading indicates polynomial predictors. The climate predictors were centred with species' range median values. P-values were calculated with Wald tests.

References for Figs. S1–S3 and Tables S2–S4

Elevatr: Access elevation data from various APIs. Manual.

Fick, S. E., and R. J. Hijmans. 2017. WorldClim 2: new 1-km spatial resolution climate surfaces for global land areas. International Journal of Climatology 37:4302–4315.

Hacket-Pain, A., J. J. Foest, I. S. Pearse, J. M. LaMontagne, W. D. Koenig, G. Vacchiano, M. Bogdziewicz, et al. 2022. MASTREE+: Time-series of plant reproductive effort from six continents. Global Change Biology 28:3066–3082.

Hollister, J., T. Shah, J. Nowosad, A. L. Robitaille, M. W. Beck, and M. Johnson. 2023.

Lobry, J. R., M.-C. Bel-Venner, M. Bogdziewicz, A. Hacket-Pain, and S. Venner. 2023. The CV is dead, long live the CV! Methods in Ecology and Evolution n/a.

Mapzen. 2023. Terrain Tiles. https://registry.opendata.aws/terrain-tiles/.

Massicotte, P., and A. South. 2023. Rnaturalearth: World map data from natural earth. Manual.

Section S1

References

Allen, R. B., N. W. H. Mason, S. J. Richardson, and K. H. Platt. 2012. Synchronicity, periodicity and bimodality in inter-annual tree seed production along an elevation gradient. Oikos 121:367–376.

Barringer, B. C., W. D. Koenig, and J. M. H. Knops. 2013. Interrelationships among life-history traits in three California oaks. Oecologia 171:129–139.

Bogdziewicz, M., M. Fernández-Martínez, J. M. Espelta, R. Ogaya, and J. Penuelas. 2020. Is forest fecundity resistant to drought? Results from an 18-yr rainfall-reduction experiment. New Phytologist 227:1073–1080.

Buechling, A., P. H. Martin, C. D. Canham, W. D. Shepperd, and M. A. Battaglia. 2016. Climate drivers of seed production in *Picea engelmannii* and response to warming temperatures in the southern Rocky Mountains. Journal of Ecology 104:1051–1062.

Espelta, J. M., P. Cortés, R. Molowny-Horas, B. Sánchez-Humanes, and J. Retana. 2008. Masting mediated by summer drought reduces acorn predation in Mediterranean oak forests. Ecology 89:805–817.

Fernández-Martínez, M., J. Belmonte, and J. Maria Espelta. 2012. Masting in oaks: Disentangling the effect of flowering phenology, airborne pollen load and drought. Acta Oecologica 43:51–59.

Kelly, D., D. E. Hart, and R. B. Allen. 2001. Evaluating the wind pollination benefits of mast seeding. Ecology 82:117–126.

Kelly, D., M. H. Turnbull, R. P. Pharis, and M. S. Sarfati. 2008. Mast seeding, predator satiation, and temperature cues in Chionochloa (Poaceae). Population Ecology 50:343–355.

LaMontagne, J. M., M. D. Redmond, A. P. Wion, and D. F. Greene. 2021. An assessment of temporal variability in mast seeding of North American Pinaceae. Philosophical Transactions of the Royal Society of London. Series B, Biological Sciences 376:20200373.

Lázaro, A., A. Traveset, and M. Méndez. 2006. Masting in Buxus balearica: assessing fruiting patterns and processes at a large spatial scale. Oikos 115:229–240.

Le Roncé, I., J. Gavinet, J.-M. Ourcival, F. Mouillot, I. Chuine, and J.-M. Limousin. 2021. Holm oak fecundity does not acclimate to a drier world. New Phytologist 231:631–645.

Masaki, T., S. Abe, S. Naoe, S. Koike, A. Nakajima, Y. Nemoto, and K. Yamazaki. 2020. Horizontal and elevational patterns of masting across multiple species in a steep montane landscape from the perspective of forest mammal management. Journal of Forest Research 25:92–100.

Mencuccini, M., P. Piussi, and A. Z. Sulli. 1995. Thirty years of seed production in a subalpine norway spruce forest patterns of temporal and spatial variation. Forest Ecology and Management.

Mooney, K. A., Y. B. Linhart, and M. A. Snyder. 2011. Masting in ponderosa pine: comparisons of pollen and seed over space and time. Oecologia 165:651–661.

Pearse, I. S., J. M. LaMontagne, M. Lordon, A. L. Hipp, and W. D. Koenig. 2020. Biogeography and phylogeny of masting: do global patterns fit functional hypotheses? New Phytologist 227:1557–1567.

Roland, C. A., J. H. Schmidt, and J. F. Johnstone. 2014. Climate sensitivity of reproduction in a mast seeding boreal conifer across its distributional range from lowland to treeline forests. Oecologia 174:665–677.

Smaill, S. J., P. W. Clinton, R. B. Allen, and M. R. Davis. 2011. Climate cues and resources interact to determine seed production by a masting species. Journal of Ecology 99:870–877.

Sullivan, J. J., and D. Kelly. 2000. Why is mast seeding in Chionochloa rubra (Poaceae) most extreme where seed predation is lowest? New Zealand Journal of Botany 38:221–233.

Tanentzap, A. J., W. G. Lee, and D. A. Coomes. 2012. Soil nutrient supply modulates temperature-induction cues in mast-seeding grasses. Ecology 93:462–469.

Webb, C. J., and D. Kelly. 1993. The reproductive biology of the New Zealand flora. Trends in Ecology & Evolution 8:442–447.

Wion, A. P., I. S. Pearse, K. C. Rodman, T. T. Veblen, and M. D. Redmond. 2023. Masting is shaped by tree-level attributes and stand structure, more than climate, in a Rocky Mountain conifer species. Forest Ecology and Management 531:120794.

Wion, A. P., P. J. Weisberg, I. S. Pearse, and M. D. Redmond. 2020. Aridity drives spatiotemporal patterns of masting across the latitudinal range of a dryland conifer. Ecography 43:569–580.

Section S2

Below, we compare our covered gradients compared to reported species limits (or the limits of species' presence) for the 6 species with the most data. We acknowledge that these reported species limits (predominantly based on Joint Research Centre et al. (2016)) contain uncertainties. Yet, they indicate that the gradients sampled in our analysis are broadly representative.

Table S5: Species climate limits compared with our sampling gradient

Species	Variable	Source of limits	Limits	Sampled
Fagus sylvatica	Temperature (MAT, degree celcius)	Hacket-Pain et al. 2016	Low: ~ 6.5<br>High: 13.5	Low: 2.7<br>High: 12.9
		Joint research centre et al. 2016	Low: ~ 3<br>High: 14	
	Precipitation (AP, mm)	Hacket-Pain et al. 2016	Low: ~ 500<br>High: 1272	Low: 620<br>High: 1694
		Joint research centre et al. 2016	Low: ~ 500<br>High: ~ 1500	
Picea abies	Temperature	Joint research centre et al. 2016	Low: ~ -3<br>High: ~ 11	Low: -1.7<br>High: 10
	Precipitation	Joint research centre et al. 2016	Low: ~ 450<br>High: ~ 2500	Low: 520<br>High: 1446
Pinus edulis	Temperature	Burns 1990	Low: 4<br>High: 16	Low: 5.7<br>High: 13.6
	Precipitation	Burns 1990	Low: 250<br>High: 560 (locally 690)	Low: 270<br>High: 539
Picea glauca	Temperature	Thompson et al. 2023	Low: - 10<br>High: 6	Low: -4.8<br>High: 6.5
	Precipitation	Burns 1990	Low: 250<br>High: 1270	Low: 274<br>High: 1185
Pinus sylvestris	Temperature	Joint research centre et al. 2016	Low: ~ -3<br>High: ~ 14	Low: -2.3<br>High: 11.6
	Precipitation	Joint research centre et al. 2016	Low: 400<br>High: 2500	Low: 397<br>High: 1864
Quercus robur/petraea	Temperature	Joint research centre et al. 2016	Low: 5<br>High: 15	Low: 3.4<br>High: 12.5
	Precipitation	Joint research centre et al. 2016	Low: ~ 500<br>High: ~ 1900	Low: 601<br>High: 1166

References for Section S2

Burns, R. M. 1990. Silvics of North America: Conifers. U.S. Department of Agriculture, Forest Service.

Hacket-Pain, A. J., L. Cavin, A. D. Friend, and A. S. Jump. 2016. Consistent limitation of growth by high temperature and low precipitation from range core to southern edge of European beech indicates widespread vulnerability to changing climate. European Journal of Forest Research 135:897–909.

Joint Research Centre (European Commission), J. San-Miguel-Ayanz, D. De Rigo, G. Caudullo, T. Houston Durrant, and A. Mauri. 2016. European atlas of forest tree species. Publications Office of the European Union.

Thompson, R. S., K. Anderson, R. T. Pelltier, L. E. Strickland, S. Shafer, and P. J. Bartlein. 2023. A gridded database of the modern distributions of climate, woody plant taxa, and ecoregions for the continental United States and Canada. U.S. Geological Survey.
